# Mobilising climate action with moral appeals in a smartphone-based 8-week field experiment

**DOI:** 10.1038/s44168-025-00282-x

**Published:** 2025-08-28

**Authors:** Viktoria Spaiser, Nicole Nisbett

**Affiliations:** School of Politics and International Studies, University of Leeds, Leeds, UK

**Keywords:** Climate-change mitigation, Interdisciplinary studies

## Abstract

Effective climate change mitigation requires profound lifestyle changes and citizens’ support for transformational climate policies. We present a comprehensive, highly granular, field-experiment dataset of people’s self-reported, daily, real-life behaviours measured in CO_2_e across six domains, as well as their civic and political behaviour. The data (*N* = 156, 7615 repeated observations over 8 weeks) was collected via a bespoke smartphone app and is enriched by people’s daily reflections on their change trajectories and by data on political leaning, emotions, agency, socio-demographics, values, attitudes and social norms. The study shows that exposing people to moral appeals results in overall carbon footprint reduction (particularly from heating, food and consumption), and in greater civic and political climate action, including among people leaning politically to the centre and right. However, the treatment could lead to some backlash, i.e. increased carbon footprint (particularly from food and car journeys) in people who hold egoistic values.

## Introduction

Climate change mitigation requires aligned transformations across multiple sectors and domains of social, economic and political life^1^, which includes lifestyle and behaviour change^2^. According to estimates by the UK Climate Change Committee, at least 32% of emission reductions by 2035 rely upon individuals’ and households’ behavioural changes^3^. The importance of people’s actions is even greater if behavioural change includes changes in civic and political behaviour, e.g. how people vote^2^. Despite some concerns that individual climate mitigation behaviour may crowd out climate mitigation policy support^4^, research suggests that in fact there is a strong correlation between the two^5–7^, not least because once behavioural change has been practiced, it is perceived as more feasible, which increases domain-matched policy support^8^.

Survey research has suggested that while many people are concerned about climate change and want governments to do more to stop global warming, the enthusiasm to change lifestyles or to support policies that would lead to lifestyle changes is much lower^9–12^. Various studies have investigated specific behaviours in various domains, typically focussing on single behaviours (e.g. energy demand^13,14^, diet^15^, transport^16,17^, tree planting^18^) and how these behaviours could be changed through a range of interventions, based on social norms^19,20^, appeals to moral norms^14,21^, highlighting co-benefits^22,23^, making people reflect their choices before nudging^15^, real-time feedback^24^, combining boosting (enhancing behavioural competencies) and nudging^25,26^, promoting the emergence of pro-environmental social identities^27^, visioning^17^, carbon literacy^28,29^ or financial incentives^30^. Where behavioural change was achieved, the effects were often small^18^, but the effect sizes vary significantly with study and intervention designs^30^. Furthermore, many studies are detached from people’s real everyday lives^31^, but where real-life behaviour (e.g. offsetting of flights) has been researched, behavioural interventions can be quite effective^32^.

Few studies examined behaviours more holistically^29^, seeking to change lifestyles rather than specific behaviours. Staats et al., for instance, studied over three years, though not continuously, a set of real-life environmental consumer behaviours and how it was affected by an intervention combining information, feedback and social interaction^33^. However, the focus was on relatively low-effort environmental behaviours (e.g. shower duration) rather than more impactful behaviours that would signify lifestyle changes (e.g. shifting to a plant-based diet). Moreover, relatively little attention has been given to civic and political behavioural change beyond donations^34^ and studying climate activists’ mobilisation^35,36^. It is rarely included in (experimental) multidimensional environmental behavioural studies, though survey studies have demonstrated correlations between consumer environmental behaviour and civic/political climate action^5,37^. And Lauren et al. showed experimentally, albeit solely with a sample of mostly female students, that reminding people of their past (low-effort) pro-environmental behaviour spills over at least into intentions for pro-environmental civic/political engagement^38^. Noteworthy are also non-experimental, cross-country studies that assess a range of predictors and their effects on a range of behaviours, including policy support and pro-environmental behaviour tasks. Interestingly, while most predictors showed divergent patterns, only a couple of predictors, including internal environmental motivation, had consistent effects across outcomes^39^.

**Fig. 1:**
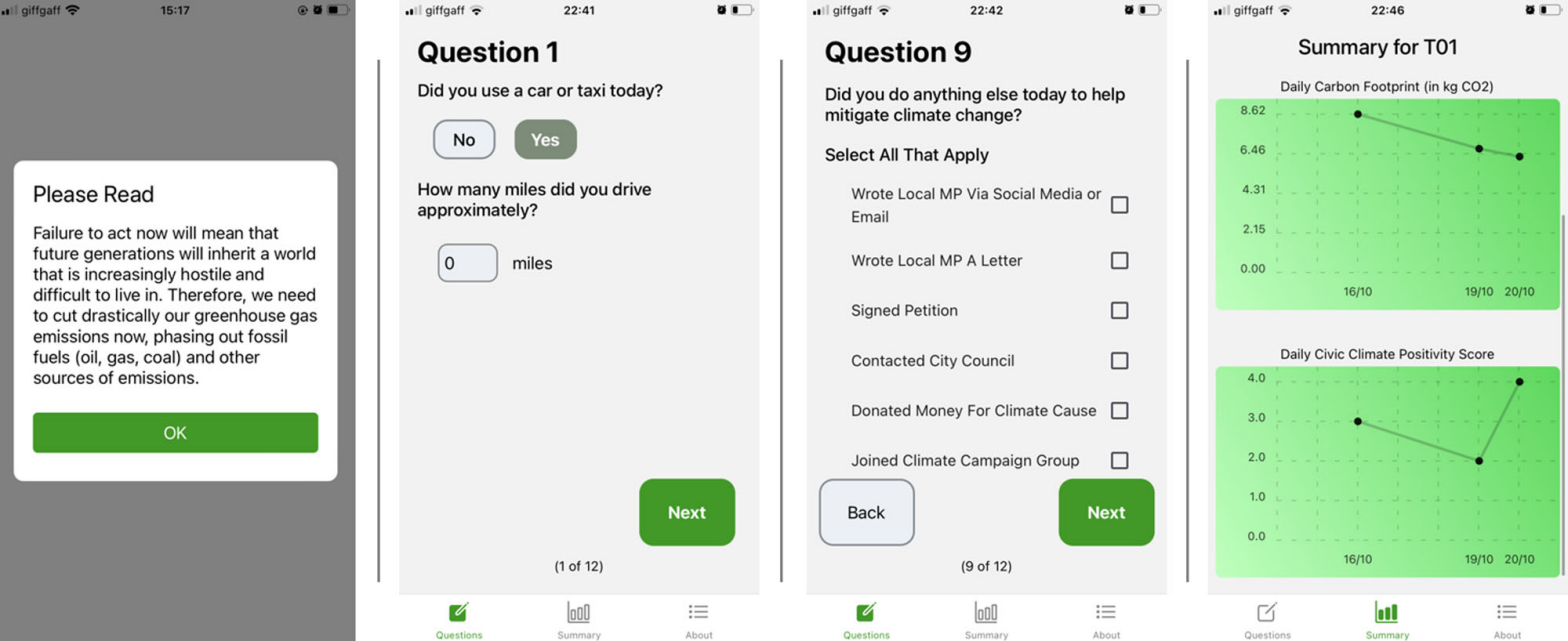
Screenshots from the Climate Champ app, used in the study to collect data and deliver the intervention in the treatment group. The first image shows one example message shown daily to study participants in the treatment group, revealing the moral implications of fossil fuels. The second image shows an example question to estimate the carbon footprint from daily activities, here from car journeys. The third image shows an example question to estimate the civic climate positivity score from daily civic/political climate action. The fourth image shows the visual display of a study participant’s carbon footprint and civic climate positivity score (see “Methods” for further details).

This pre-registered randomised control design study examines the effect of moral appeals over multiple real-life behavioural domains of significance for climate change mitigation. The daily self-reported behaviours included car usage, flying, energy demand, heating, diet and non-grocery purchasing of new items; all measured with high temporal granularity in CO_2_e units. Furthermore, included was information behaviour, talking about climate change, and various civic and political actions such as contacting political representatives. This behavioural data was enriched further by qualitative data from people’s daily behavioural and attitudinal reflections. Capturing multiple behaviours allows us to study differences in people’s behavioural responses; behaviour might be relatively easier and quicker to change in some domains (e.g. reducing electricity demand) than in others (e.g. shifting diet to plant-based). We developed a bespoke mobile phone app, Climate Champ (see Fig. 1), to conduct the study, which demonstrates how carbon-tracking apps can be utilised in a field-experimental design to study people’s real-life climate-affecting behaviour, incl. civic and political climate action. The study ran for eight weeks to capture potential temporal dynamics (e.g. learning) of behavioural change. With 156 study participants recruited by a marketing research agency from the general UK population, the dataset contains a total of 7615 observations across a range of behavioural dimensions (see “Methods” for further methodological and sampling details).

Following the Capability, Opportunity and Motivation Model for Behavioural Change (COM-B Model)^40^, we presented every day behaviour as an *opportunity*, while aiming at manipulating the *motivation* component with our treatment. The visualisation of participants’ individual carbon footprint and civic and political action through the app (see Fig. 1) offered near-time feedback to help participants, motivated to change their behaviour, to learn how they can do it (e.g. learning which activities lead to a higher carbon footprint), ensuring minimum *capability*. To manipulate the motivation component, participants in the treatment group were exposed to daily varying, pretested^41^ moral appeals, which they would see when opening the app (see Fig. 1). We note that the moral appeals were AI-generated, as the pre-test study suggested people found AI-generated moral statements more convincing. Participants in the control group would not see any message when opening the app. These moral appeals were based on Moral Foundations Theory^42^, as moral foundations concerning care/avoiding harm and fairness were found to be positively associated with people’s willingness to contribute to climate change mitigation^43–45^. Specifically, a moral argument on the basis of a moral foundation such as care (e.g. “If we take action on climate change, it will send a powerful signal to our children and grand-children that we care about their future and are willing to make sacrifices for them”) was given as a reason for why “we need to drastically cut our greenhouse gas emissions now, phasing out fossil fuels (oil, gas, coal) and other sources of emissions.” The intervention establishes an explicit link between fossil fuels as the main driver of climate change through GHG emissions and the moral foundations of care and justice that demand cutting these emissions by phasing out fossil fuels. Moral foundations underlie internalised moral norms^43^, where violation results in feelings of guilt^46^. Indeed, research suggests that guilt enhances the persuasive effects of moral norms^47^. This intervention was also chosen because several studies demonstrated the power of activating people’s moral norms for behavioural change^16,48,49^. In one of the largest studies (60,000 people across 23 countries in Europe, North and South America, Middle East, Africa, Asia and Australia) to date on how to mobilise climate action, Marshall et al. found that a framing emphasising the responsibility to protect the planet for future generations (care moral foundation) was 12x more persuasive across the world than a framing emphasising green jobs, opportunities and economic growth^50^. This finding is also supported by other studies^51,52^ that found that responsibility towards future generations is a strong predictor of pro-environmental engagement. Here, we investigate how effective such moral appeals are in changing people’s lifestyles and civic/political engagement.

## Results

### Reducing carbon footprint

**Fig. 2:**
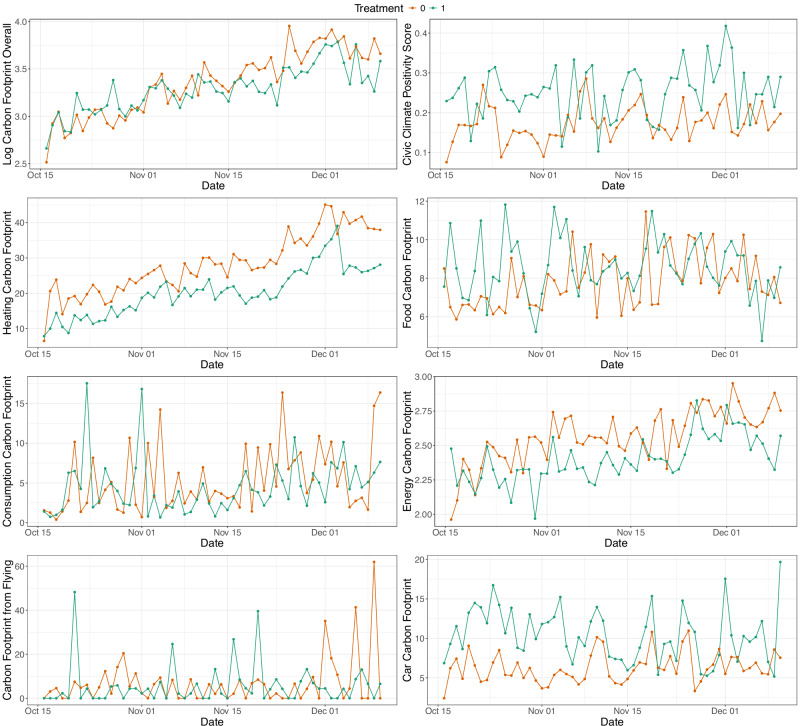
Aggregate mean lines of the carbon footprint overall and across the domains, as well as the civic climate positivity score in the two groups.

**Fig. 3:**
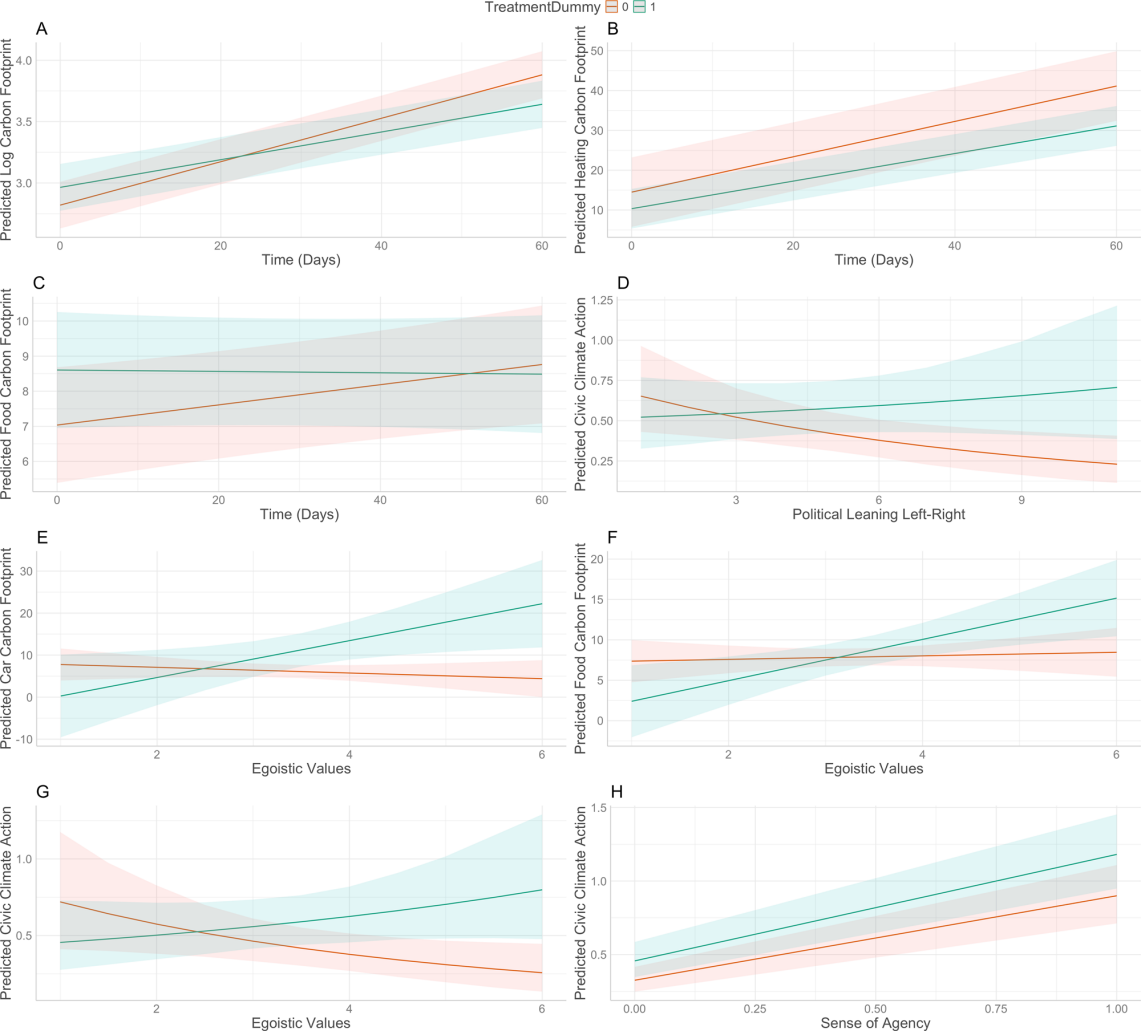
Interaction plots for interaction fixed effects. Interaction plots for interaction fixed effect between treatment and time on log carbon footprint (**A**), heating carbon footprint (**B**), food carbon footprint (**C**), between treatment and political leaning on civic climate positivity score (**D**), between treatment and egoistic values on car journeys carbon footprint (**E**), food carbon footprint (**F**), civic climate positivity score (**G**) and cross-level interaction between treatment and sense of agency on civic climate positivity score (**H**).

We first tested the effect of the treatment on the overall carbon footprint, i.e. the first main, pre-registered hypothesis: Exposure to moral arguments for climate action decreases an individual’s carbon footprint. We log-transformed the measure in response to the extreme skewness and range of the data (see “Methods”). Running a set of Mixed Effect Models and using the likelihood ratio test (LRT) to identify significant fixed effects and best-fit models (see Supplementary Table 2.1), suggests that our treatment had indeed an effect, but only in interaction with time (see Table 1, Fig. 3A), which captures the upwards trend in the data (see Fig. 2). The upwards trend is down to the season (mid-October to mid-December 2023) during which data was collected, which required people to increasingly heat their homes etc. as we will discuss below. However, the increase in carbon footprint was flatter in the treatment group, resulting in an overall lower carbon footprint in this group. The predicted daily difference is about 19kgCO_2_e (converting the log-scale back to CO_2_e) at the end of the study between the two groups (see Fig. 3A).

**Table 1:** Mixed-effects models for treatment and time fixed effects

Outcome variable	Best model	LRT	FE (treatment)	FE (time– date)	FE (interaction)
Log carbon footprint overall	Mixed effect model (random intercept) with interaction between time and treatment fixed effect	base: 490.83, *p* < 0.001 with time FE only: 23.709, *p* < 0.001	0.145 (−0.12, 0.42)	0.018 (0.016, 0.02)	−0.006 (−0.009, −0.004)
Heating carbon footprint	Mixed effect model (random intercept, treatment random slope), with interaction between time and treatment fixed effect	base: 728.19, *p* < 0.001 no interaction: 11.07, *p* = 0.001 no random slope: 25.11, *p* < 0.001	−4.148 (−13.9, 6.77)	0.444 (0.40, 0.49)	−0.098 (−0.15, −0.04)
Food carbon footprint	Mixed effect model (random intercept, treatment random slope), with interaction between time and treatment fixed effect	base: 32.568, *p* < 0.001 no interaction: 6.4963, *p* = 0.039^a,^ no random slope: 25.884, *p* < 0.001	1.578 (−0.89, 3.82)	0.029 (0.006, 0.05)	−0.031 (−0.06, 0.004)
Consumption carbon footprint	Mixed effect model (random intercept, treatment random slope), treatment & time fixed effects	base: 20.9, *p* = 0.0003 no time FE: 9.28, *p* = 0.002	−1.04 (−3.08, 1.07)	0.063 (0.02, 0.10)	NA
Electricity carbon footprint	Mixed effect model (random intercept), time fixed effect	base: 120, *p* < 0.001	NA	0.007 (0.006, 0.01)	NA
Carbon footprint from flying	Base model (random intercept)	NA	NA	NA	NA
Car carbon footprint	Mixed effect Model (random intercept, treatment random slope), treatment fixed effect	base: 68.484, *p* < 0.001 no random slope: 64.135, *p* < 0.001	4.670 (0.55, 9.10)	NA	NA
Civic climate positivity score	Zero-inflated Poisson mixed effect model (random intercept), treatment & time fixed effects, time fixed effect for excess zeros	base: 67.65, *p* < 0.001 with random slope no time FE: 56.36, *p* < 0.001	0.293 (0.04, 0.55)	0.008 (0.006, 0.01) zero-part coefficient: 0.003	NA

Note that analyses with domain-specific outcomes are explorative. Full details of all models in Supplementary Tables 2.1, 2.2, 2.3, 2.4, 2.5, 2.6, 2.7, 2.8.

*LRT* likelihood ratio test, against the base model and, where applicable, against the next best model(s) that were better than the base model.

base: base mixed effect model with just random intercept.

*FE* fixed effect.

95 % Confidence interval in brackets.

Bonferroni-corrected alpha value for confirmatory (pre-registered) tests is 0.002 and 0.008 for exploratory (non-pre-registered) tests. ^a^ Models that do not pass the Bonferroni-corrected alpha value.

*N* = 7615 (156 participants, 56 days).

For greater interpretability of the results, we disaggregated the overall carbon footprint into separate domains to explore (not pre-registered) the treatment effect in separate domains. For the carbon footprint from heating, we can establish an independent treatment fixed effect (see Supplementary Table 2.2), but the best model is again one that includes an interaction between treatment and time fixed effect, while also including random slopes (see Table 1). The interaction (see Fig. 3B) shows that the seasonal trend of increased carbon footprint from heating was again flatter in the treatment group. Including random slopes suggests important individual variance in response to the treatment. Inspecting the random slope values suggests that the treatment effect in the treatment group ranges between −2.44 and −11.49 kgCO_2_e. Qualitative data from daily reflective comments of study participants suggests that people perceive more behavioural control when it comes to reducing heating carbon footprint, which may partially explain why the treatment effect is particularly pronounced in this domain. A treatment group participant, for instance, wrote: “I went to put heating on this morning but put a jumper on instead. I did still have heating on, but only for 1 h rather than 2.” An additional motivator here could be the cost saving, as indeed some comments from participants have suggested, but this motivator would be equally strong in both groups.

When it comes to food carbon footprint from consuming meat and dairy products, the treatment has an effect, but again only in interaction with time (see Table 1, Fig. 3C) and including random slopes (treatment). The interaction suggests that the food carbon footprint started to decline just very slightly in the treatment group, a few weeks into the study, while in the control group, the food carbon footprint started to rise, potentially linked to pre-Christmas time when the traditional diet becomes richer in meat and dairy. The comments from study participants suggest that many needed some time to learn how much carbon footprint their diet was generating, and only then did some decide to reduce their meat and dairy intake. One study participant, for instance, wrote one month into the study: “I’ve found reducing meat intake reduces my carbon footprint far more than I expected, so I’m going to continue to work on this.” It should also be noted that people often explore dietary changes gradually, which does not necessarily translate into changes on a daily basis, e.g. one participant wrote: “I plan to not eat meat or dairy products for half of this week to help reduce my carbon footprint.” Individual variation, captured through the random slopes, was moreover remarkably large. While many participants in the treatment group were willing to decrease their food carbon footprint (on average by between −0.24 and −6.05 kgCO_2_e daily), some did not respond to the treatment and maintained their meat/dairy-rich diet (two outliers, i.e. repeatedly very high meat/dairy intake in the treatment group). This complexity in behavioural response likely contributes to the uncertainty (potential for false positive) we note for food carbon footprint model outcomes.

The LRT test (see Table 1) suggests that the intervention also had an effect on reducing carbon footprint from non-grocery purchases of new things in the treatment group by a daily average of -1.04 kgCO_2_e. However, the confidence interval, which includes a zero, weakens this conclusion, as the uncertainty about the effect seems rather big. While including time as a fixed effect improves the model further, no significant interaction effect between treatment and time was measurable. The slight upward trend in both groups from mid-November is likely due to people buying Christmas presents. Though some participants were speaking of their desire to do Christmas shopping more sustainably, one participant, for instance, wrote: “We are looking at buying second-hand Christmas presents to reduce waste.” The model with random intercepts and random slopes is moreover the better model, which points again to the importance of individual variation when it comes to behavioural response. The estimated average daily reduction of carbon footprint in the treatment group varied between −0.27 and −16.22 kgCO_2_e, though for a few, an increase in CO_2_e (around +1 kg CO_2_e) was also estimated.

No significant difference between the treatment and control group was measurable with respect to electricity carbon footprint and carbon footprint from flying (see Table 1). The significant trend upwards in electricity carbon footprint over time is likely seasonal, as people spend more time indoors in colder months, needing more light, relying more on tumble dryers, etc. Electricity demand reduction seems difficult to achieve, probably because high electricity prices in the UK mean that people are already trying to save energy^53^. Participants reported their attempts to reduce their energy demand, but it did not translate into measurable patterns in the data. E.g. one participant in the treatment group wrote: “We continue to keep our large appliance usage to a minimum. Opting for fewer cycles on the dishwasher and washing machine. It seems to have resulted in a decline in our energy usage.” There are other avenues for reducing electricity carbon footprint, such as switching to energy-efficient appliances, forgoing extra appliances (e.g. drinks fridge) or timing heavy electricity usage to coincide with times when the carbon intensity of the grid is lowest. Such lifestyle changes are often episodic, and to capture the carbon footprint effects from electricity demand timing would require a different data collection strategy. We have captured some episodic lifestyle changes (e.g. switching to a greener energy provider) and report them in the last paragraph of this section. Some participants have also mentioned timing their heavy electricity usage, but we do not have a systematic account of such behaviours. Future research should, however, attempt to capture such behavioural changes more systematically.

With respect to flying, the best model was the base model, with neither the treatment nor time having any effect (see Table 1). However, data for this domain was very sparse, as ordinary people do not fly frequently. Furthermore, the effects would need more time to materialise as people book their flights typically weeks in advance. What we noticed from comments, though, is how some participants were shocked when realising the carbon footprint that their flights have generated, for instance, a participant from the treatment group said: “I’m flying to NYC this weekend. For the first time, I checked what the carbon impact is. Is it me, or does the following suggest we need to ban commercial air travel. From myclimate.org: My flight: 6.9t CO_2_. To stop climate change, this is the maximum amount of CO_2_ that can be generated by a single person in a year.” And some participants reported of how they changed their holidays plans to avoid flying. But we could also observe some moral licensing at the individual level^54^, where individuals justify “immoral” behaviour (e.g. flying) by having previously engaged in “moral” behaviour (e.g. reduced meat/dairy intake). For instance, a participant in the control group, who otherwise showed great motivation in reducing their carbon footprint (e.g. heating, diet, civic action) wrote: “I’m happy because I’m on holiday. Yes, I’ve taken a flight; however, I feel that I deserve it. The flight was full, and it would have been gone even if I wasn’t on board; therefore, I feel like there’s nothing I could have done.”

An unexpected finding is that the moral appeal intervention appears to have the effect of increasing carbon footprint from car usage in the treatment group, with time having no significant effect (see Table 1). This is the only domain where we observe an effect opposite to what we expected. As the mixed effect model with random intercept and random slopes is the best model, the effect size and direction vary strongly (ranging between −4.718 and +80.83 kgCO_2_e) between study participants in the treatment group. This suggests that individual circumstances and/or characteristics played an important role in shaping behavioural response to the treatment. Indeed, for over half (42) of the participants in the treatment group, the intervention had the predicted negative effect, i.e. lowering their carbon footprint from driving on average by −3.34 kgCO2e daily. And comments from study participants in the treatment group testify that quite a few attempted to reduce their car usage, e.g. E17: “I’m heading into London today using the train instead. More expensive, but a decision made in the interest of reducing dependence on fossil fuels.” But, for 36 participants, the effect was positive with extreme variations between +0.54 and +80.83 more kgCO_2_e. The randomisation in the creation of the two groups has produced comparable groups across a range of socio-demographic and attitudinal variables (see Supplementary Note 1). However, we cannot rule out that despite the randomisation, there were some differences between the two groups that affected car usage, e.g. by chance, more participants in the treatment group needed to commute long distances for work. For instance, participant E01, one of the outliers in the treatment group with respect to driving carbon footprint, wrote: “I’m driving a lot of miles for work, I work as a clinician in the NHS, but I work across the county. I also have a terminally ill relative who lives far away, which was where the big mileages come from”. Comments also suggest that people perceived less behavioural control in this domain. One study participant, for instance, wrote, “I was working today, so had to take a taxi as public transport is very unreliable.” An alternative interpretation could be that people perceive less behavioural control in domains where they failed to change their behaviours consistently, i.e. lack of behavioural control could be a post-hoc rationalisation of one’s own behaviour^55,56^. However, research shows that in domains such as transport, people (in the UK) have indeed often limited agency due to lock-ins and structural constraints^57^. Yet another explanation for this unexpected finding is a covariate that interacts with the treatment, as we will discuss below.

We note also episodic behavioural changes, which were not included in the carbon footprint estimations but will be significant in the long run. For instance, two study participants from the treatment group reported that they purchased or started leasing an electric vehicle toward the end of the study; several other study participants also said they were looking into purchasing an electric vehicle. Study participants made inquiries into or started green retrofitting their homes during the study, e.g. one participant from the treatment group insulated their loft and external walls after researching green retrofit options at the start of the study. Several study participants, mostly from the treatment group, were planning to install air heat pumps and/or solar panels, but high upfront costs are for many an inhibiting factor. Some participants, again mostly from the treatment group, reported changing their electricity supplier for a greener option and changing to a greener bank and pension fund that commit to fossil fuel divestments.

### Mobilising civic and political climate action

The moral appeal intervention had a significant effect (see Table 1) on civic and political climate action, confirming the second main, pre-registered hypothesis: Exposure to moral arguments for climate action increases an individual’s civic climate positivity score. Time also had a significant, independent positive effect, which may suggest a learning effect facilitated by the app design (see Table 1). Exponentiating the treatment fixed effect to obtain the incidence rate ratio suggests that participants in the treatment group had a 34% higher civic and political climate action incidence rate. That is, participants in the treatment group were more likely to engage in some form of civic and political climate action, and they were more likely to engage in several civic and political climate actions on the same day. The actions ranged from seeking information on climate change and climate solutions, talking to others about climate change and climate solutions and taking political action like joining a climate campaign group. We note, however, that the overall level of daily engagement was relatively low. On average, participants would record only 17 (16 in the control group, 18 in the treatment group) out of 56 days at least one civic/political climate action (see also Supplementary Fig. 2.3).

Individual variance seemed to play a lesser role here than in the models for carbon footprint, but individual variation played a role in the type of activities participants chose to engage with. The data show a preference for seeking information (including checking banks’/pensions’ climate credentials) on climate change and climate change solutions, and for talking to others about climate change and climate solutions. Participants also reported signing petitions, making donations and joining online groups on climate change. Several have joined climate campaign groups and climate action events, initiated climate action at work and contacted their councils. Fewer participants reached out to their MPs. One participant from the treatment group, for instance, wrote: “I am looking to email my MP to protest against the new drilling licences. I am very anti-new fossil fuel licences in any form.” Most study participants in the treatment group (54.9%, compared to 45.1% in the control group) pledged moreover to vote for a party with ambitious climate policies at the next general election. Finally, eight participants reported joining climate protests; two of them, both from the treatment group, who had not attended climate protests before, did so repeatedly.

Previous research emphasises the importance of talking to others about climate change and solutions, something confirmed by our study. One participant, for instance, said: “I met up with friends today and I told them about the climate study I’m taking part in. They were really interested, and we ended up watching videos on YouTube about climate change. I told my friends about the climate pledge I made, where I have a minimum of 2 meat-free days per week, and they thought that was a great idea, as it’s very easy to incorporate, and they pledged to do it too”. This shows the potential for diffusion of behavioural change^32,58,59^.

### Covariates

The fact that the best models for carbon footprint in certain domains (heating, food, non-grocery consumption, and car usage) required the inclusion of random slopes suggests that individual variation played an important role. We therefore investigated several covariates to understand which individual characteristics could explain some of the individual variations. Table 2 summarises the influence of two covariates that proved particularly important (see Supplementary Note 3 for full model details for all tested covariates).

**Table 2:** Mixed effect models with added covariates, political leaning, and egoistic values

Outcome variable	Best model	LRT	FE (covariate)	FE (treatment)	FE (interaction)
Heating carbon footprint	Best model (see Table 1) + egoistic values fixed effect	5.33, *p* = 0.021^a^	−4.680 (−8.78, −0.78)	−3.563 (−13.30, 7.29) 0.444 (0.40, 0.48) −0.098 (−0.15, −0.04)	NA
Food carbon footprint	Best model (see Table 1) + political leaning fixed effect	8.342, *p* = 0.004	0.632 (0.20, 1.08)	1.411 (−0.90, 3.72) 0.029 (0.01, 0.05) −0.031 (−0.06, 0.001)	NA
Food carbon footprint	Next best model (see Table SI 2.2, M3) + treatment/egoistic values interaction FE	5.057, *p* = 0.024^a^	0.218 (−0.72, 1.27)	−7.300 (−14.3, −0.50)	2.333 (0.38, 4.41)
Car carbon footprint	Best model (see Table 1) + political leaning fixed effect	7.085, *p* = 0.008^a^	0.913 (0.28, 1.59)	4.424 (0.09, 9.00)	NA
Car carbon footprint	Best model (see Table 1) + treatment/egoistic values interaction FE	6.108, *p* = 0.040^a^	−0.673 (−2.23, 0.87)	−12.557 (−27.70, 1.76)	5.066 (0.89, 9.50)
Civic climate positivity score	Best model (see Table 1) + treatment/political leaning interaction FE	6.35, *p* = 0.042^a^	−0.094 (−0.18, −0.01)	−0.187 (−0.80, 0.42)	0.118 (−0.001, 0.24)
Civic climate positivity score	Best model (see Table 1) + treatment/egoistic values interaction FE	7.43, *p* = 0.024^a^	−0.232 (−0.43, −0.04)	−0.855 (−1.73, 0.02)	0.362 (0.11, 0.62)

Note that analyses with domain-specific outcomes and with egoistic values are explorative. Full details of all models in Supplementary Tables 3.1, 3.2, 3.3, 3.4, 3.5, 3.6, 3.7.

*LRT* likelihood ratio test, showed for the test against the respective best model in Table 1.

*FE* fixed effect; for food carbon footprint and heating carbon footprint, the interaction FE between treatment and time is shown (first estimate for treatment, second for time, third for interaction).

95% Confidence interval in brackets.

Bonferroni-corrected alpha value for confirmatory (pre-registered) tests is 0.002, 0.006 for exploratory (non-pre-registered) tests with egoistic values as covariate (across all outcome variables) and 0.008 for political leaning as covariate across domain-specific carbon footprints as outcome variables. ^a^ Models that do not pass the Bonferroni-corrected alpha value.

*N* = 7615 (156 participants, 56 days).

In the pre-registered hypothesis six, we assumed that individuals who identify as rather on the political left will have a lower carbon footprint and a higher civic climate positivity score and that this effect will be stronger pronounced in the experimental group. The hypothesis was not pre-registered for the domain-specific carbon footprints, though; the models for these are hence explorative. While political leaning, self-placement on a political left-right scale (higher values are more politically right), has no effect on the overall carbon footprint (see Supplementary Note 3), it explains some of the individual variance from car usage and dairy/meat consumption footprint without interacting with the treatment. Individuals more on the right of the political spectrum have a higher carbon footprint from car journeys and from dairy/meat consumption in both groups. We found, however, an interaction effect between treatment and political leaning for civic and political climate action (see Fig. 3D). The interaction suggests that there is not much difference between very left participants in both groups, possibly because they already engage in some form of civic/political climate action. The effect of the treatment becomes more prevalent the more we move to the centre and right. We note that this result is the opposite of what we expected in our pre-registered hypothesis six.

We also found that egoistic values explain some of the individual variation. Importantly, taking egoistic values into account explains at least partially the paradoxical effect of the treatment on carbon footprint from car journeys. There is an interaction effect. While the treatment does result in a lower car journey carbon footprint among those who do not hold egoistic values, the treatment seems to result in a greater carbon footprint in those who hold egoistic values (see Fig. 3E). A similar interaction effect (see Fig. 3F) can be measured for food carbon footprint. In both domains, the treatment seems to lead to a backlash amongst those holding egoistic values. Interestingly, egoistic values seem to result in a lower carbon footprint from heating, irrespective of the treatment, possibly because of cost-saving incentives. We also find an interaction effect between treatment and egoistic values for the civic climate positivity score (see Fig. 3G), which suggests that there is little difference between the two groups for people who reject egoistic values. The treatment effect becomes pronounced as we move toward the mean (3.34, SD = 1.02) and above, making those with average and higher levels of egoistic values more likely to engage in some civic or political climate action as they are exposed to the treatment. Maybe it is those with a more egoistic outlook who need reminding of their moral obligation to engage in some climate action, and as they reject behavioural change that is inconvenient or inconsistent with their image of a successful, influential individual^60^, they might at least feel they must engage in some form of civic or political action. However, these are tentative results based on explorative analyses. They confirm some previous findings on egoistic values^61^, but also introduce some potential nuance to be further studied. For instance, if the civic action involved seeking information on climate change, they may seek or encounter climate denial/delay information. And indeed, some study participants reported this, e.g. one participant wrote: “I read the Greenpeace co-founder Dr Patrick Moore suggests that human-induced global warming is a complete fabrication; his article was a very interesting read”.

We note that most models, including covariates do not pass the Bonferroni-corrected alpha value (see Table 2). We acknowledge the potential for false positives with respect to these results, on the other hand correcting for false positives comes at the cost of increasing the likelihood of false negatives. We therefore report these largely exploratory results here, to inspire further investigations of these findings.

A noteworthy, time-varying covariate is perceived agency with respect to climate change. Using a panel regression model, we found that it correlates both at the within (0.77, 95% CI: 0.70, 0.85) and between (1.31, 95% CI: 0.84, 1.77) level with civic and positive climate action (see Supplementary Table 3.9). This means perceived agency explains to some extent the difference between participants but also the day-to-day difference within individuals, with a stronger sense of agency corresponding with more climate action. We also found a significant cross-level interaction effect (0.21, 95% CI: 0.06, 0.36, see Fig. 3H) between sense of agency and treatment that suggests the treatment effect on civic and political climate action was reinforced by a sense of agency (i.e. steeper increase in climate action as we move toward higher values of sense of agency in the treatment group). Sense of agency did, all in all, not play a significant role with respect to carbon footprint (see Supplementary Note 3.5).

Emotional state (time-varying covariate, see Supplementary Note 3.6), social norm perceptions (see Supplementary Table 3.6) and climate change worry (see Supplementary Table 3.7) were found not to explain individual variation in carbon footprint or civic and political climate action. Though the emotional state had some minor within-level effects and was itself affected by the treatment, with participants in the treatment group more likely to report negative emotions (*X*^2^ = 67.85, *p* < 0.001, Supplementary Note 3.6). Perception of anti-fossil fuel norms, while having no effect on the behavioural outcome, was also affected by the treatment (Diff = −0.34 [95% CI: −0.60, −0.08], *t* = −2.59, *p* = 0.01, see Supplementary Note 3.3). People in the treatment group perceived anti-fossil fuel norms as being or becoming more established at the end of the study, compared to the start of the study, before they were exposed to any treatment, which was meant to reveal the moral implications of fossil fuels. This result supports the effectiveness of moral appeals that reveal the moral implications of fossil fuels.

With respect to socio-demographic covariates (see Supplementary Notes 3.5 and 3.6), no noteworthy correlations or interactions were found, though having children seems to act as a motivator for civic and political climate action, which is also confirmed by comments from participants, who reported conversations with their children about climate change (solutions). Finally, while outside the scope of this paper, we note that we also conducted some preliminary testing into spillover effects between behaviours (see Supplementary Note 4) and some preliminary analysis of individual change trajectories (see Supplementary Note 5). Future analyses could build on these preliminary results.

## Discussion

This study demonstrates that people, including those leaning more to the political right, respond to moral arguments for climate action that are built on moral foundations around care (avoiding harm) and fairness by embracing some lifestyle changes and becoming active climate citizens. This aligns with previous findings suggesting that moral foundations based in care and fairness can be effective across the political spectrum^41,44,62,63^. These results also align with the COM-B Behavioural Change Model^40^, as we demonstrate that behavioural change can be induced by manipulating motivation, presenting everyday behaviour as an opportunity and facilitating minimum capability through the app design. However, our study proposes to consider integrating the Moral Foundation Theory^42^ into the COM-B model. We show that moral arguments based in the Moral Foundation Theory can be a powerful motivator for behavioural change, something not explicitly acknowledged by the COM-B Model, which sees morality as personal standards of right and wrong^64^, rather than as societally shared foundations that likely serve as a stronger motivator than merely personal standards, even partially overcoming counteracting values (e.g. egoistic values). Our study also suggests that the effect of moral appeals does not wear off; we did not see a drop in the treatment effect over time.

We acknowledge the limitations of our study. We rely on self-reported behaviours, and we cannot rule out that seeing the moral appeals resulted in “recalling” more climate action in the treatment group. However, if that were the case, we would expect to see more consistent treatment effects, but we record much more nuanced responses to the treatment, including no effect in some domains and even an opposite effect in one domain. Currently, there are no other, more reliable means of collecting behavioural data with that granularity. While carbon-tracker apps such as Cogo use financial transactions logged on a banking account to estimate the carbon footprint of their users, this method can be flawed. Important information (e.g. what exactly was purchased) is missing for an accurate estimate. Still, if spend-based carbon footprint estimations become more accurate, they may be an alternative in future studies.

Future studies should also aim to increase sample size and duration and to include other interventions to compare the effectiveness of various interventions (e.g. different types of appeals) as well as implement a difference within difference field-experiment design, collecting pre- and post-intervention data with a control and experiment group condition. A difference-in-difference design would also help to control for possible effects resulting from the app design (e.g. feedback effect from visualisation), which we have not explicitly measured in our study. Though we note that the time covariate does capture some effects, such as learning which behaviours reduce carbon footprint (i.e. developing capability) from observing one’s own carbon footprint trend visualised through the app.

Finally, this study also provides some insights into the domains where most policy intervention is needed to allow people to transition towards climate-positive behaviour. The transport sector is particularly in need of interventions to increase access to good public transport, with behavioural change being unlikely without such investments. Participants also stated the need for financial support for green retrofitting. Deep lifestyle changes do indeed often rely on systems change^65^, but it is also the citizens who need to demand these system changes, and our study shows that moral appeals can help mobilise citizens to make these political demands.

## Methods

### Sample

We conducted a pre-registered two-group repeated-measures field-experiment study, building on a previous pilot^66^. The study lasted for 8 weeks (16th of October to 10th of December 2023). The 156 adult (at least 18 years old) study participants were recruited by People for Research (study participant recruitment agency) from the general UK population, representing various demographics. Participants did not self-select; rather, they were selected by People for Research from the company’s own pool of potential study participants, using quota sampling based on UK census data to ensure the sample is as representative of the UK population as possible. Six participants dropped out of the study within the first two weeks and were replaced by new participants recruited by People for Research, to ensure the pre-registered minimum sample size of 150. The sample size decision was based, on the one hand, on budget constraints and on the other hand on power estimates (see pre-registration^67^ for details). Study participants were randomly (simple randomisation) assigned to two equally sized groups, the control group and treatment group (see Supplementary Note 1 for breakdown of socio-demographic, attitudinal and lifestyle distributions in the overall sample and in the two groups for randomisation check).

Participation was incentivised through a payment of £280 for daily participation throughout 8 weeks. Additionally, two prize draws were organised. The payment was independent of whether participants reduced their carbon footprint or engaged in climate action, but deductions were made if study participants missed submitting their data on a daily basis. Participants varied to some extent in their diligence to engage daily with the app and record their behaviour. While overall the response rate was very good (90.7%), once we exclude the six dropped-out study participants, there are occasional missing data entries, with most study participants having missed recoding their behaviour on 2–4 days over a period of 56 days.

### Research design

Ethical approval for the study was granted (0743) by the Business, Environment, Social Sciences (BESS + FREC) Ethics Committee, University of Leeds, UK.

Participants in the treatment group would see a daily varying notification with a moral argument for climate action to encourage climate-positive behaviour (see Fig. 1, see pre-registration^67^ for full list of messages), when opening the app Climate Champ. They would see the message whenever opening the app. The messages were pre-tested in terms of how convincing they were perceived to be^41^. To avoid habituation due to repetition, there were a total of 28 messages. Given the duration of the study (8 weeks, 56 days), study participants in the experimental group would see each message twice over the duration of the entire study. There was no random shuffling of messages; all experimental group participants would see the same message on any given day. Participants in both groups were not explicitly told that they were in the experimental or control group and were equally instructed in the pre-study briefing that they should try to reduce their carbon footprint and engage in various climate action activities.

The engagement with the Climate Champ app itself—recording daily climate-affecting behaviour and seeing the personal carbon footprint and civic climate positivity score visualised—could have served as an indirect nudge to reduce greenhouse emissions. However, as app interaction was held constant in both groups and as we measure differences between the control and experimental group, we are confident that the effects we measure can be attributed to the moral appeals in the notifications seen by participants in the experimental group. But, in future, a difference-in-difference study design could help to better disentangle the moral appeal effects from a possible app usage effect. Furthermore, while we cannot entirely rule out that the notification itself, irrespective of its content, could have influenced self-reported behaviour, we believe this to be unlikely, as previous studies suggest that information alone is not sufficient to affect behavioural change^68^. Moral appeals, on the other hand, were suggested to be effective in previous studies^14,16,21,48^. Furthermore, we found that treatment group participants’ perception of anti-fossil fuel social norms changed significantly (in comparison to the control group), i.e. they perceived anti-fossil fuel norms as more socially established after participating in the study, in comparison to pre-participation (see Supplementary Note 3.3).

To mitigate issues with self-reporting, we primed study participants at the start of the study to report accurately and honestly and get in touch with us if they were unsure. Moreover, given the 8-week period of daily data collection, daily over- or under-reporting is likely to average out. Generally speaking, research suggests greater reliability of self-reported diary data, as used in our study, in comparison to one-shot questionnaires, as people do not have to struggle to recall what they did^69^. We note that the changes in behaviours (e.g. changes in carbon footprint) are calculated from the self-reported behaviours (see next paragraph); they are not estimated by the study participants themselves.

### Measures

At the start of the study the recruited study participants completed an online survey (see pre-registration^67^ for questionnaire), where they were asked to provide socio-demographic information (age, gender, living conditions (rural or urban and whether they own the property they are inhabiting), household income, education, ethnicity, first three digits of their postcode) and answer questions on their values (altruistic, biospheric, hedonistic, egoistic), social norm perceptions, climate change worry and political leaning (left-right scale self-placement). After that, the study participants needed to set up a user account on the app Climate Champ, which we specifically developed for the purpose of this study and which is available under public license on GitHub (https://github.com/ViktoriaSpaiser/climate-champ-app-public). When setting up their user account, participants had to complete three set-up questions to calibrate the calculation of the individual carbon footprint: (1) what type of car they own (if any), (2) what type of heating they use in their property and (3) their average daily electricity usage (see pre-registration^67^ for all app questions). Once they were set up, study participants were required to answer a set of questions (about 10 min) on the app every day in the evening (the app was programmed to allow behavioural recording from 16:00 to 23:59), including whether they were using a car (and how many miles, see Fig. 1), whether they were flying (and how many hours), whether they were consuming meat/dairy/fish products, whether they were purchasing listed new non-grocery items, how much energy they were using that day and whether they were heating their homes (and for how many hours).

The answers to these questions (calibrated by the answers from the three setup questions) were used to estimate the domain-specific as well as the overall carbon footprint (in kgCO_2_e), based on carbon footprint estimates provided in Berners-Lee^70^ (see pre-registration^67^ for specific calculations/scoring). To provide an example: if a study participant answered on a given day to the question “Did you use a car or taxi today?” with “Yes” and specified that they drove 12 miles and we know from their account setup that they drive a SUV/4 × 4 car, then their answer is translated and recorded within the app database as 12 * 1.26 = 15.12 kgCO_2_e. According to Berners-Lee a SUV/4 × 4 car generates on average 1.26 kgCO_2_e per mile, comparing to 0.53 kgCO_2_e per mile when driving an average petrol car. The 15.12 kgCO_2_e is the car usage carbon footprint of that study participant on that given day. Similarly, answers to questions with respect to heating, diet, non-grocery consumption, flying and electricity usage were translated into respective kgCO_2_e. For the overall carbon footprint, the domain-specific carbon footprints were added up and it is the overall carbon footprint that is visualised through the app (see Fig. 1). For statistical analysis purposes the overall carbon footprint was log-transformed to reduce impact from outliers (e.g. when carbon footprint shoots up because a participant took a flight) and to reduce skewness in the data (long tail distribution with few, but very high carbon footprint values due to outliers) and approximate normality (see Supplementary Fig. 1.11).

Furthermore, study participants were asked to report whether they spoke to various people (e.g. family, friends, colleagues) about climate change and what their reaction was, whether they obtained any information about climate change from various sources (e.g. TV, newspaper, social media) and whether they did any of the listed actions (e.g. contact MP, join climate campaign group, see Fig. 1) to mitigate climate change (see pre-registration^67^ for all app questions). Based on the answers to these three questions, a daily civic climate positivity score was calculated, essentially a count variable, summing up the distinct civic and political climate actions. For instance, if a study participant recorded on a given day that they looked for climate change information online, that they also spoke to their colleague about climate change and signed a petition demanding climate action from the government, their civic climate positivity score would add up to three on that given day.

Study participants were also asked how they felt emotionally (angry, anxious, content, happy, sad, stressed, see Supplementary Fig. 3.2) on the given day and whether they thought they had any agency when it comes to climate change (see Supplementary Fig. 3.1). There was also one open text question at the end, where participants could report anything else they deemed relevant or reflect on their actions. This open-text option proved quite important to make sense of the data collected via the app, as study participants made use of this option to explain and reflect on their behaviours.

At the end of the study, study participant had to complete a final online survey to evaluate their overall experience with the study and reflect on their trajectory of change. We also used this final survey to check people’s voting intentions with respect to climate change and to check whether any changes in attitudes, social norm perceptions or values could be recorded after study participation in comparison to the attitudes recorded during the initial survey (see pre-registration^67^ for final survey questionnaire). For a detailed breakdown of all variables, see pre-registration^67^.

### Analysis

The two main pre-registered hypotheses tested here are:

**H1:** Exposure to moral arguments for climate action decreases an individual’s carbon footprint.

**H2:** Exposure to moral arguments for climate action increases an individual’s civic climate positivity score.

The first hypothesis was tested for the overall carbon footprint as pre-registered and exploratively (not pre-registered) for the carbon footprint in distinct domains, i.e. carbon footprint from driving, flying, heating, diet, consumptions and electricity. The explorative analyses were added because disaggregating the effect for various domains provides more nuanced insights.

Pre-registered hypothesis **H3** was formulated for a combined (overall carbon footprint and civic climate positivity score) index of climate positive behaviour and was tested too (see Supplementary Note 2.9), with the same outcomes as for overall log carbon footprint. However, given the nuances in behavioural responses, using a combined index appears to be less suitable. For that reason, the results were not included in the main manuscript.

We also tested most of the secondary pre-registered^67^ hypotheses **H4–H18**, which mostly concern the effects of time and various covariates. Not tested were hypotheses H12, H15 and H16 due to sparse data (H15, H16) or because the analyses would go beyond the scope of this paper, requiring a different methodological approach (H12). See Supplementary Note 6 for a complete overview of all pre-registered hypotheses and a summary of results for each hypothesis. We note that we did not pre-register hypotheses with respect to the effect of egoistic values (or hedonic values), as the pre-registered hypotheses were only formulated for altruistic (H7) and biospheric values (H8). Thus, the discussed effects with respect to egoistic values are based on explorative analyses.

We used linear Mixed Effect Models (MEMs) to test hypotheses with respect to log carbon footprint and carbon footprints in distinct domains as the outcome variables. Repeated-measures data inherently involve non-independence, as observations from the same subject are correlated. MEMs are specifically designed to account for this hierarchical data structure by modelling both fixed effects (population-level effects, like the intervention) and random effects (subject-specific variability, which captures the correlation within subjects), thereby obtaining unbiased parameter estimates and valid standard errors^71^. The models with the treatment dummy variable (fixed effect) were tested with a Likelihood Ratio Test (LRT) against a base (null) model with just random intercepts. LRT was also used to compare models of various additional specifications (e.g. including time fixed effects, including interaction term between time and treatment, etc.) to identify the best model. Significant treatment effects were thus deduced from the LRT. P-values for coefficients are not reported because MEMs do not allow for meaningful coefficient p-value interpretation, as parameters are not independent, a requirement to compute the F-statistics^72^. Additionally, an individual p-value only tests the significance of one parameter conditional on all others being in the model. It does not assess the overall contribution of a set of related parameters (e.g. the main effect of an intervention and its interaction with time), nor does it provide a direct comparison of the overall fit between two competing model structures (e.g. a model with the intervention effect vs. one without it). This can lead to misleading conclusions or difficulty in establishing the overall importance of the intervention. The LRT is a powerful and principled method specifically designed to compare two nested models, where one model (the null hypothesis) is a simpler version of the other (the alternative hypothesis). To test the effect of an experimental intervention, one typically compares a “full” MEM that includes the intervention term (and any relevant interactions) against a “reduced” MEM that excludes these terms. The null hypothesis is that the more complex model (with the intervention) does not significantly improve the fit over the simpler model (without the intervention). This method hence provides a more direct, global test of whether the intervention significantly improves the model’s ability to explain the observed data^73^. While the asymptotic *χ*2 distribution for the LRT in MEMs theoretically relies on a high number of subjects, the large number of observations (7615) generated through repeated measurement, generally helps to stabilise the likelihood estimation and leads to reasonable performance even with a moderate number of subjects, especially when compared to the less reliable approximations for individual p-values in complex MEMs^74,75^. Finally, while we included bootstrap confidence intervals for the fixed effect estimates, these too should be interpreted cautiously in Mixed Effect Models. Models were estimated using the R package lme4^76^.

To test hypotheses with respect to civic and political climate action as the outcome variable a zero-inflated poisson mixed effect model was used to account for the fact that the civic climate positivity score produced lots of zero values (when a participant did not engage in any civic/political climate action on a given day) and that the dependent variable was a count variable, adding up the activities the participants engaged in. Otherwise, the same procedure was followed as above to determine the best model. Models were estimated using the R package GLMMadaptive^77^.

To account for the increased likelihood of false positives due to multiple hypothesis testing and multiple outcomes, we have applied the conservative Bonferroni Correction to the Likelihood Ratio Test, accounting for the two main pre-registered outcomes (overall carbon footprint, civic climate positivity score) and the 15 tested pre-registered hypotheses. Applying this correction to the pre-registered alpha value of 0.05 results in a new alpha value of 0.05/(15 * 2) = 0.002. A separate Bonferroni Correction was applied to the models with additional outcomes (six domain-specific carbon footprints), investigating exploratively the treatment effect, with the new alpha value being 0.05/6 = 0.008. A separate Bonferroni Correction was also applied to the models with all outcome variables (eight) exploring the effect of the covariate egoistic values, with the new alpha value being 0.05/8 = 0.006. We treat the pre-registered and non-pre-registered (exploratory) statistical analyses as distinct sets for the purpose of multiple hypothesis correction, because of the fundamental difference in their inferential goals and the associated control over the Type I error rate. Pre-registered hypotheses constitute a confirmatory set of analyses. By preregistering, researchers commit to testing a specific, pre-defined set of hypotheses using a pre-specified analytical plan before observing the data. The primary goal here is hypothesis testing, aiming to make strong, inference-valid claims. In this context, applying a multiple hypothesis correction (e.g., Bonferroni) to this specific set of pre-registered tests ensures that the family-wise error rate— the probability of making at least one Type I error across all tests in this family—is controlled at the desired alpha level (e.g. *α* = 0.05). This maintains the statistical rigour and reduces the chance of false positives. Conversely, non-pre-registered exploratory analyses are typically performed after observing the data, driven by curiosity, unexpected findings, or a desire to uncover additional patterns. Their primary goal is hypothesis generation and descriptive insights, rather than direct confirmation. These analyses involve a certain risk of “p-hacking”, where numerous tests might be implicitly or explicitly run until a statistically significant result is found by chance. If a single multiple comparison correction were applied across both pre-registered and exploratory analyses, it would unfairly penalise the confirmatory power of the pre-registered tests for the sake of the opportunistic exploratory ones. By treating the exploratory analyses as a separate “family,” the integrity of the confirmatory phase is maintained, while transparently acknowledging the hypothesis-generating nature and greater uncertainty associated with the exploratory findings, which would ideally require independent replication for confirmation. We also note that there is trade-off when using multiple hypothesis testing correction, as it increases the Type II error rate^78–80^.

Panel regression models, and specifically within-between effect models, were used to include time-varying covariates, i.e. sense of agency and emotional states, both of which were recorded daily along with the behavioural outcomes. In these models we also included socio-demographic covariates (see Supplementary Notes 3.5 and 3.6). Models were estimated using R package panelr^81^.

Finally, we conducted some first tests on behavioural spillovers (see Supplementary Note 4), where behaviour change is accompanied by subsequent changes in other behaviours related to the same goal (e.g. climate mitigation)^82^. These analyses were not the focus of this paper, but they could be expanded in the future.

## Supplementary information

Supplementary Information re2

## Data Availability

The data is published along with the pre-registration on OSF: 10.17605/OSF.IO/Y78CU. R Markdown files and respective compiled html files are published along with the pre-registration and data on OSF: 10.17605/OSF.IO/Y78CU. R version 4.4.2 was used.
