# Supplementary material for: Mobilising climate action with moral appeals in a smartphone-based 8-week field experiment
Source: NPJ Clim Action. 2025 Aug 28;4(1):81. doi: 10.1038/s44168-025-00282-x (PMC12394071; doi:10.1038/s44168-025-00282-x)
Supplement: Supplementary file 1 — Supplementary Information re2 [file 44168_2025_282_MOESM1_ESM.pdf]

Supplementary Information

Table of Contents

Supplementary Note 1: Data Description *3*

Supplementary Note 1.1: Socio-Demographics 3

Supplementary Note 1.2: Political Leaning 5

Supplementary Note 1.3: Values 6

Supplementary Note 1.4: Climate change worry 8

Supplementary Note 1.5: Social Norms 9

Supplementary Note 1.6: Carbon Footprint Calibration Variables 10

Supplementary Note 1.7: Log-Transformation Overall Carbon Footprint 11

Supplementary Note 2: Mixed Effect Models - Full Model Information *11*

Supplementary Note 2.1: Overall Carbon Footprint 12

Supplementary Note 2.2: Heating Carbon Footprint 13

Supplementary Note 2.3: Food Carbon Footprint 14

Supplementary Note 2.4: Non-Grocery Consumption Carbon Footprint 15

Supplementary Note 2.5: Electricity Carbon Footprint 16

Supplementary Note 2.6: Flying Carbon Footprint 17

Supplementary Note 2.7: Car Journeys Carbon Footprint 18

Supplementary Note 2.8: Civic Climate Positivity 19

Supplementary Note 2.9: Models with Climate Positive Behaviour Index 20

Climate Positive Behaviour Index 1 20

Climate Positive Behaviour Index 2 22

Supplementary Note 3: Covariate Models *24*

Supplementary Note 3.1: Political leaning 24

Supplementary Note 3.2: Values 25

Altruistic Values 25

Biospheric Values 26

Egoistic Values 27

Hedonic Values 28

Supplementary Note 3.3: Social Norms Perceptions 29

Supplementary Note 3.4: Climate Change Worry 30

Supplementary Note 3.5: Panel Models with time-varying covariate sense of agency and time-in varying socio-demographic covariates 31

Log Carbon Footprint 32

Civic Climate Positivity Score 32

Car Journeys Carbon Footprint 33

Food Carbon Footprint 33

Heating Carbon Footprint 34

Electricity Carbon Footprint 34

Non-Grocery Consumption Carbon Footprint 35
Flying Carbon Footprint 35

Supplementary Note 3.6: Panel Models with time-varying covariate emotional state....36
Log Carbon Footprint 37
Non-Grocery Consumption Carbon Footprint 38
Civic Climate Positivity Score 39

Supplementary Note 4: Spillover Effect.................... 40

Supplementary Note 5: Some selected individual trajectories of change 41

Supplementary Note 6: Overview over pre-registered hypotheses.................... 43

Supplementary References 44

Supplementary Note 1: Data Description

Supplementary Note 1.1: Socio-Demographics

Although the data is not representative, the company People for Research was tasked to provide a sample of study participants that resembles as closely the UK population as possible. The following data was collected through an initial survey with study participants. The **age** of the study participants ranges between 18 (the minimum age allowed) and 70. The distribution is displayed in Figure 1.1. The figures also shows that the treatment group has on average slightly older participants, moreover the range in the treatment group is somewhat larger ranging from 18 to 70 (outlier). However, a two-sample t-test suggests that the difference in the means (37.9 years in the control group and 40.2 years in the treatment group) is not significant (t = -1.203, p = 0.231)

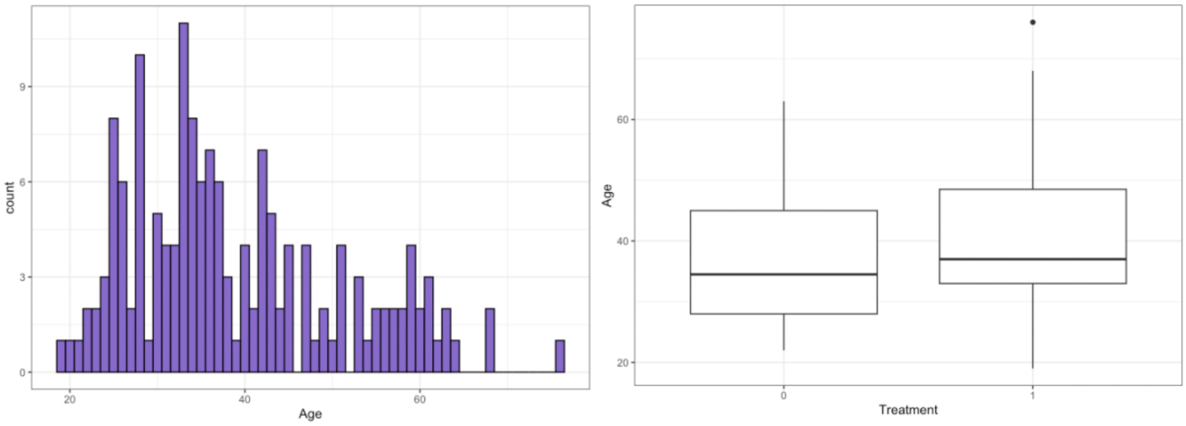

Supplementary Figure 1.1 Age distribution in overall sample, and boxplots for age distribution in the two groups, control and experimental group.

Gender is evenly distributed. 50.6% of the participants were male, 46.8% female, 1% was trans-male and 1% non-binary. This distribution is replicated in the two groups. In the control group 50.0% were male, 47.4 female, 1.3% trans-male and 1.3% non-binary. In the treatment group 51.3% were male, 46.2% female, 1.3% trans-male and 1.3% non-binary.

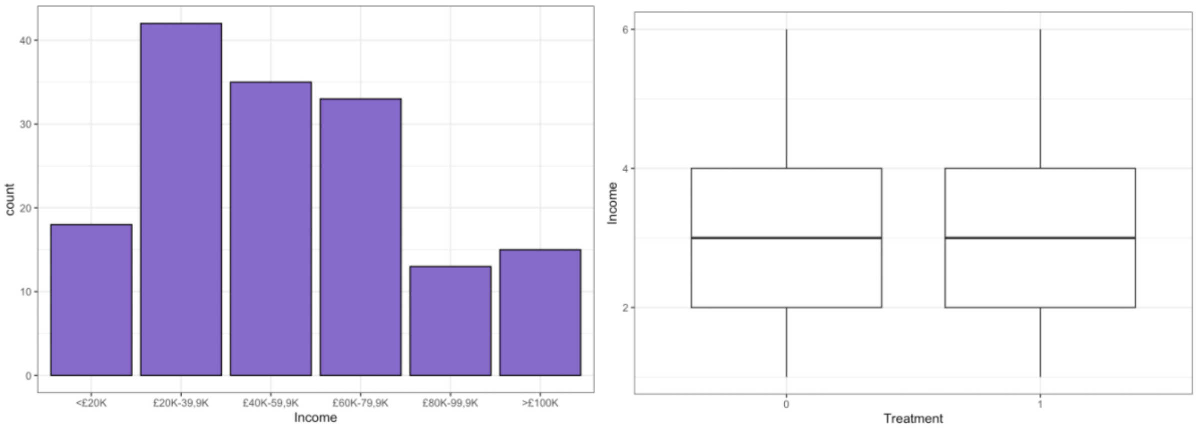

Supplementary Figure 1.2 Income distribution in overall sample, and boxplots for income distribution in the two groups, control and experimental group. Please note the income brackets are mapped to ordered numeric categories in the boxplot displays.

The **household income** distribution (Figure 1.2) approximates well the distribution in the overall UK population, with a slight overrepresentation of the income bracket “£20,000 - £39,999”. The figure also shows that the household income is equally distributed in the two groups, control group

(mean of 3.23, representing income bracket “£40,000 - £59,999”) and treatment group (mean of 3.10, representing income bracket “£40,000 - £59,999).

The **ethic composition** in the UK population was well represented in the overall sample and within each group, the control and treatment group, which have a similar ethic composition (see Table SI1.1), even though the proportion of white British participants is slightly higher in the treatment group (71.9%) compared to the control group (65.4%). A z-test (z=0.86, p=0.39) for two population proportions suggests that this difference is not significant.

**Ethnicity**	**Total**	**Control Group**	**Treatment Group**
**White British**	107	51	56
**White Irish**	3	2	1
**White Other**	10	5	5
**Black Caribbean**	2	1	1
**Black African**	6	3	3
**Black Other**	2	1	1
**Indian**	7	4	3
**Pakistani**	5	4	1
**Bangladeshi**	2	1	1
**Asian Other**	3	2	1
**Arab**	1	0	1
**Mixed**	8	4	4

Supplementary Table 1.1 Ethnicity representation in the overall sample and in the two groups, control and treatment group.

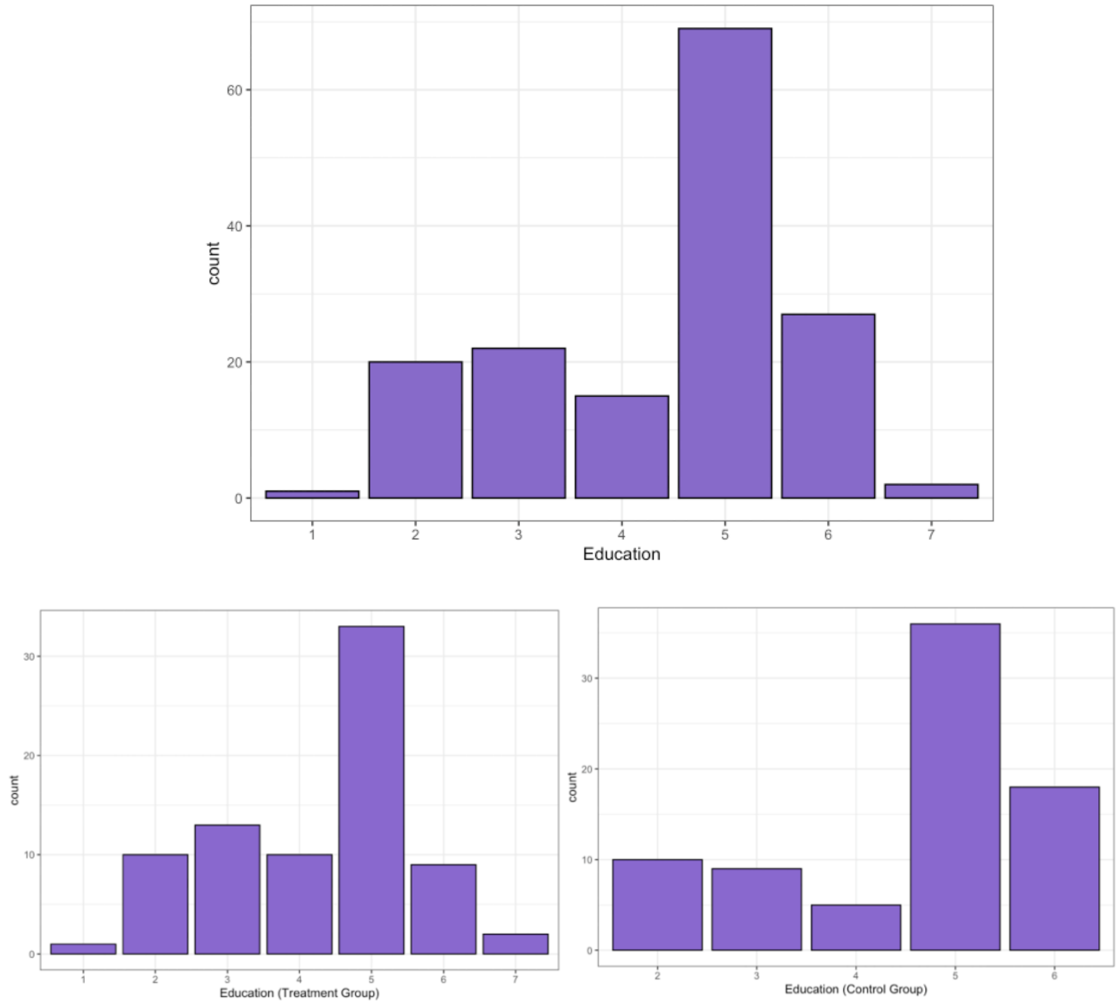

Supplementary Figure 1.3 Education distribution in overall sample, and in the two groups, control and experimental group.

Education was measured through 7 categories. The numbers 1 (No formal qualification) to 7 (PhD) can be interpreted as ordinal, with higher numbers indicating higher education. We can see that the distribution of education levels is well balanced, with 44% of participants (UK average is 40.6%) having a BA or equivalent academic degree (i.e. some form of tertiary education). We can also see some differences between our two groups (see Figure SI1.3). All education levels are represented in the treatment group, including two people with lowest level, i.e. no formal qualification and with the highest level, i.e. a PhD. This is not the case for the control group, where the lowest and highest levels are missing. On the other hand, the control group has double (18 in control group vs. 9 in treatment group) as many participants with a MA level academic degree. When looking at a dummy variable, representing those with an academic degree, i.e. a BA degree or higher, we have 62.8% of participants with an academic degree in the overall sample. In the control group 69.2% have an academic degree, while the proportion is lower in the treatment group with 56.4%. Albeit a z-test (z=1.66, p=0.097) for two population proportions suggests that this difference does not reach the significance level. Still, we will check the effect of education as a covariate.

In terms of **having children**, 44.9% of the participants in the overall sample said that they have a child or children. There is also not much difference between the two groups: in the control group 42.3% had children and in the treatment group 47.4% (z-Test, with z=0.644, p=0.522).

Area (rural/urban/suburban). Most of our study participants (81.4%) lived in urban (33.3%) or suburban areas (48.1%). This is also the case in the two groups, though there are some differences. The proportion of participants living in a rural area is somewhat higher in the control group (21.8%) than in the treatment group (15.4%). On the other hand, the proportion of people living suburban areas is higher in the treatment group (55.1%) comparing to control group (41.0%) (z-Test, with z=1.76, p=0.308), while the proportion of those living urban is somewhat lower (29.5% in the treatment group, 37.2% in the control group) (z-Test, with z=1.02, p=0.078). We created a dummy for living in a rural area or not. While the control group has a higher proportion of people living in rural areas, the difference between the two groups is not significant according to a z-Test (z=1.03, p=0.303) for proportions of two populations. Additionally fine-grained **geo-location data** (first 3-4 digits of postcode) is available.

Homeownership: The majority (53.8%) of our participants were homeowners, which means they have some agency over heating system etc. However, there are differences with that respect between the control and treatment group. In the control group only 46.2% were homeowners, while in the treatment group it was 61.5%. This difference is not significant, but just about (z=1.93, p=0.054), so we will consider homeownership dummy as a covariate. The proportions in the other categories are much more balanced, though somewhat more participants in the control group (16.7%) were living with family, comparing to participants in the treatment group (8.97%).

The **household size** ranges from 1 to 6, with one outlier (from the treatment group, E31) reporting to live in a household of 36 people (this might be a state-provided accommodation for vulnerable people e.g. homeless or refugee). The mean household size is 2.88 (sd=2.9). The two groups are comparable with respect to household hold size: treatment (M=3.13, slightly skewed due to one outlier), control (M=2.63), t=-1.066, p=0.289.

Supplementary Note 1.2: Political Leaning

With respect to political leaning on a left to right scale between 1 and 11 (rescaled from 0 to 10) with 6 representing political centre and higher values representing more right-wing political leaning, we have broad distribution with a slight skewness toward left of the centre (Figure 1.4), though the political centre position was the most chosen category (overall mean is 4.917 with standard deviation of 2.094). Figure SI1.4 also shows that the distributions with respect to political affiliation are similar in the two groups (mean in control group is 4.78, mean in treatment group is 5.05, this difference is insignificant with t=-0.80, p=0.424), albeit the treatment group is including the two outliers, who self-describe as very right.

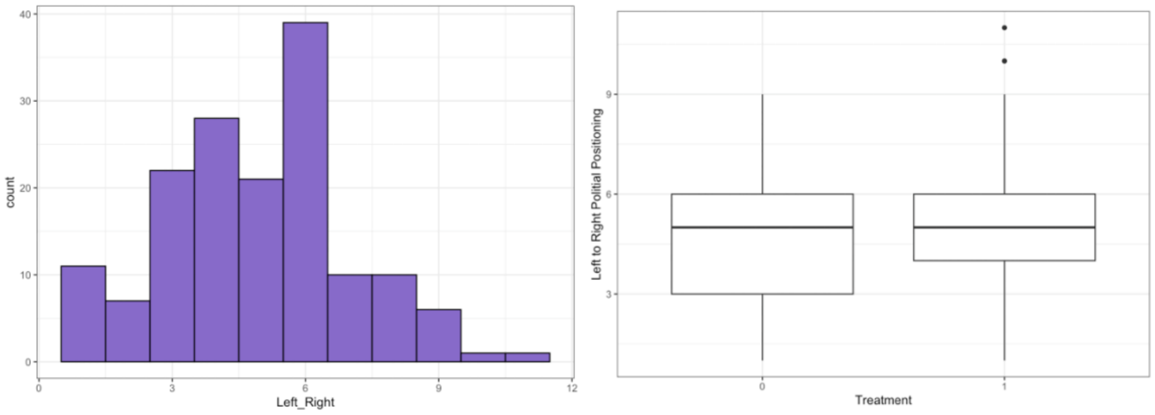

Supplementary Figure 1.4 Political leaning distribution in overall sample, and boxplots for political leaning distribution in the two groups, control and experimental group.

Supplementary Note 1.3: Values

We also explore the values that our participants hold, focusing on values that were found to be predictive of environmental behaviour in previous studies, i.e. biospheric values, altruistic values, hedonistic values and egoistic values (also self-enhancing values) (Bouman et al. 2018). Data presented here is from the initial survey, but same data was also collected through the exit survey at the end of the study and allow to investigate potential changes in values. Higher values generally meant more attuned to the respective values.

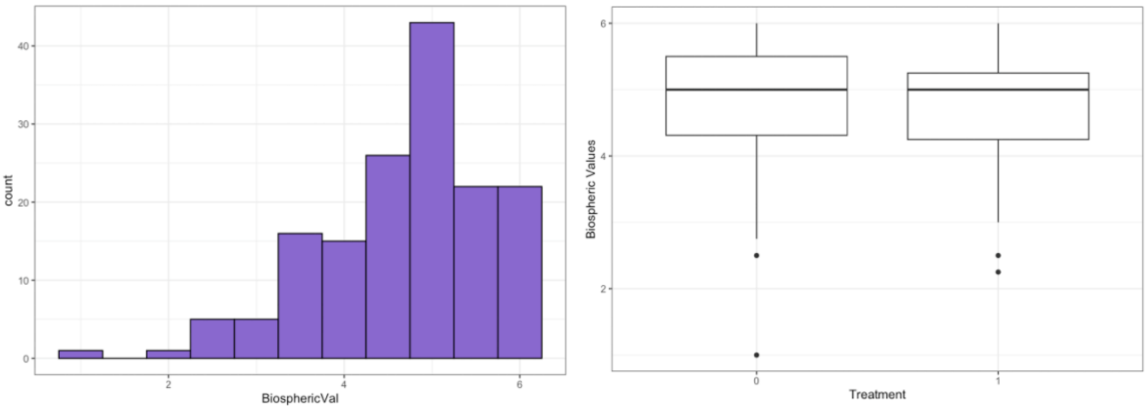

Supplementary Figure 1.5 Biospheric Values distribution in overall sample, and boxplots for biospheric values distribution in the two groups, control and experimental group.

Biospheric values were measured by asking respondents how much the descriptions below are “Not lime me at all” (1) to “Very much like me” (6). The descriptions were: (1) It is important to them to prevent environmental pollution; (2) It is important to them to protect the environment; (3) It is important to them to respect nature; (4) It is important to them to be in unity with nature. From these four items a biospheric values index was created. Biospheric values were strongly represented in the overall sample and within both groups (Figure 1.5). The overall mean is 4.803 with standard deviation of 0.957. The distributions are very similar in the two groups (mean in control group is 4.779, mean in treatment group is 4.827, t=-0.313, p=0.755). In both groups there are two outliers with rather low adherence to biospheric values.

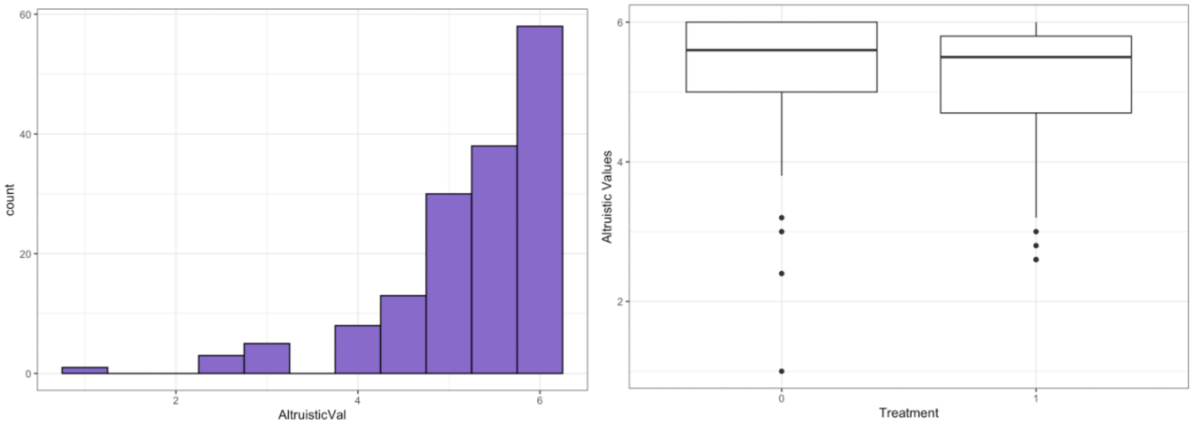

Supplementary Figure 1.6 Altruistic Values distribution in overall sample, and boxplots for altruistic values distribution in the two groups, control and experimental group.

Altruistic values were measured by asking respondents how much the descriptions below are "Not lime me at all" (1) to "Very much like me" (6). The descriptions were: (1) It is important to them that every person has equal opportunities; (2) It is important to them to take care of those who are worse off; (3) It is important to them that every person is treated justly; (4) It is important to them that there is no hate or intolerance; (5) It is important to them to be helpful to others. From these five items an altruistic values index was created. Altruistic values were again very strongly represented in the overall sample and within both groups (Figure 1.6). Higher numbers mean stronger adherence to altruistic values. The overall mean is 5.241 with standard deviation of 0.870. The distributions are very similar in the two groups (mean in control group is 5.328, mean in treatment group is 5.154, t = 1.254, p = 0.212). In both groups there are outliers (4 and 3) with rather low adherence to altruistic values.

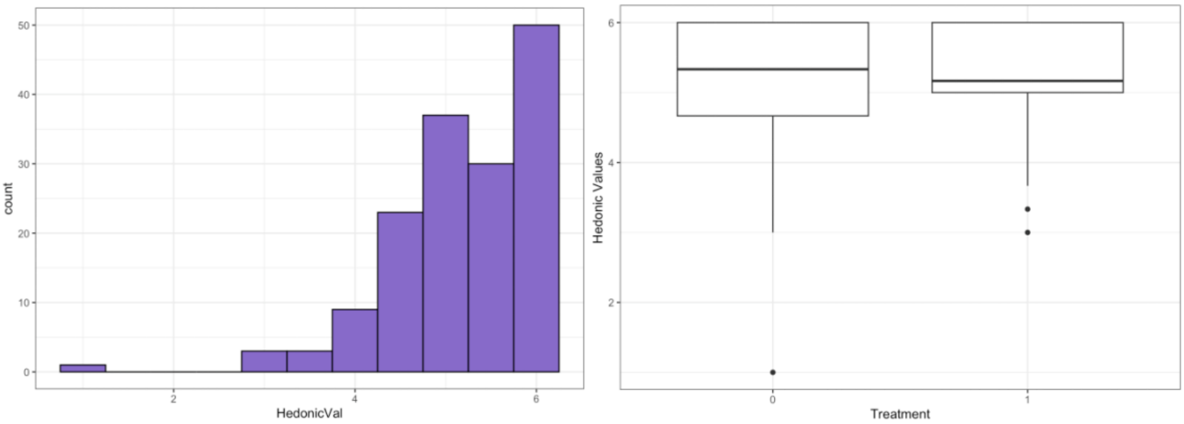

Supplementary Figure 1.7 Hedonic Values distribution in overall sample, and boxplots for hedonic values distribution in the two groups, control and experimental group.

Hedonic or hedonistic values were measured by asking respondents how much the descriptions below are "Not lime me at all" (1) to "Very much like me" (6). The descriptions were: (1) It is important to them to have fun; (2) It is important to them to enjoy the life's pleasures; (3) It is important to them to do things they enjoy. From these three items a hedonic values index was created. Hedonic values were very strongly represented in the overall sample and within both groups (Figure SI1.7), which can create value conflicts in given situations. Higher numbers mean stronger adherence to hedonic values. The overall mean is 5.197 with standard deviation of 0.801. The distributions are very similar in the two groups (mean in control group is 5.205, mean in treatment group is 5.188, t=0.133, p=0.895). In both groups there are one and two outliers with rather low adherence to hedonic values.

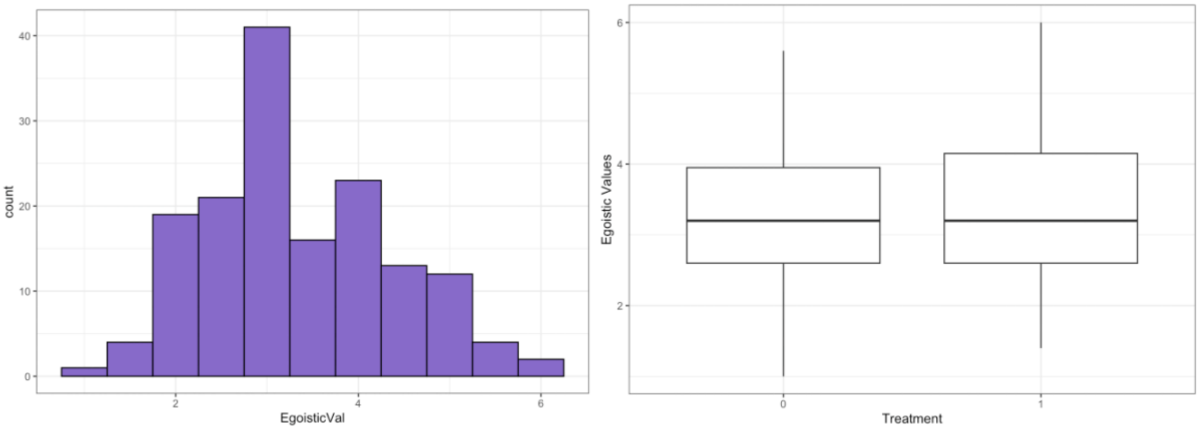

Supplementary Figure 1.8 Egoistic Values distribution in overall sample, and boxplots for egoistic values distribution in the two groups, control and experimental group.

Egoistic values were measured by asking respondents how much the descriptions below are "Not lime me at all" (1) to "Very much like me" (6). The descriptions were: (1) It is important to them to have control over others' actions; (2) It is important to them to have authority over others; (3) It is important to them to be influential; (4) It is important to them to have lots of money; (5) It is important to them to work hard and be ambitious. From these five items an egoistic values index was created. Differently from previous values with strong skewness towards higher scores, egoistic values resemble somewhat more a normal distribution (Figure SI1.8). Higher numbers mean stronger adherence to egoistic values. The overall mean is 3.358 with standard deviation of 1.038. The distributions are very similar in the two groups (mean in control group is 3.292, mean in treatment group is 3.423, t= -0.786, p=0.433).

Supplementary Note 1.4: Climate change worry

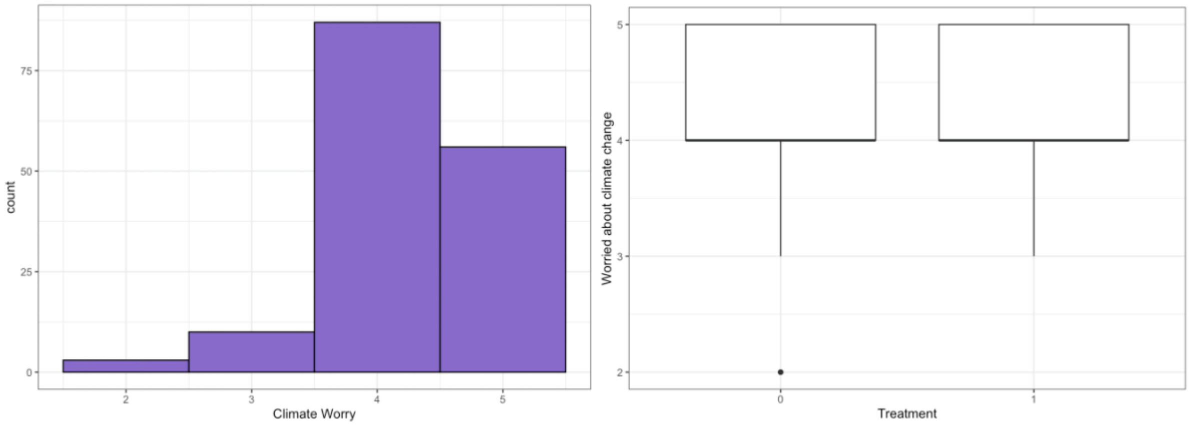

Supplementary Figure 1.9 Climate change worry attitudes distribution in overall sample, and boxplots for climate change worry distribution in the two groups, control and experimental group.

Climate change worry was measured on a scale from "Not at all worried" (1) to "Very worried" (5). Figure SI1.9 shows that no respondent said, "Not at all worried" and very few said, "Somewhat unworried" (2) or "Neither worried nor unworried" (3), with the majority picking "Somewhat worried" (4). The overall mean is 4.256 with standard deviation of 0.661. The distributions are very similar in the two groups, in fact their means are equal (mean in control group is 4.256, mean in treatment group is 4.256, t=0, p=1). In the control group we have one outlier however, with a "Somewhat unworried" response. Climate worry was assessed both through the initial survey and exist survey at the end of the study and hence changes can be assessed. The data presented here is from the initial survey.

Supplementary Note 1.5: Social Norms

We were also interested in the perception of social norms among our study participants. Again, data on norms perceptions was collected through the initial survey and the final exit survey and allows for analysis of changes. Here data from the initial survey is presented.

Norm: Is this socially acceptable?	Total	Control Group	Treatment Group
**Drive a car, even if one could easily walk the distance**			
- **No**	41%	39.7%	42.3%
- **Yes, but it is changing**	43.6%	46.2%	41.0%
**Take a flight, even if there is a good train**			
- **No**	35.9%	38.5%	33.3%
- **Yes, but it's changing**	35.3%	30.8%	39.7%
**Take a weekend flight for a European city break to go shopping or drinking**			
- **No**	17.9%	23.1%	12.8%
- **Yes, but it's changing**	27.6%	17.9%	37.2%
**Leave lights on in rooms not used**			
- **No**	71.8%	71.8%	71.8%
- **Yes, but it's changing**	18.6%	19.2%	17.9%
**Work for a fossil fuel company**			
- **No**	42.3%	42.3%	42.3%
- **Yes, but it's changing**	29.5%	29.5%	29.5%
**Invest in a fossil fuel company**			
- **No**	52.6%	50.0%	55.1%
- **Yes, but it's changing**	31.4%	30.8%	32.1%
**Vote for a political party that plans to expand national oil & gas extraction**			
- **No**	55.8%	51.3%	60.3%
- **Yes, but it's changing**	25.0%	23.1%	26.9%
**Talk about climate change at a social event**			
- **Yes**	72.4%	70.5%	74.4%
- **No, but it's changing**	10.9%	12.8%	8.97%
**Campaign for ambitious climate change policies**			
- **Yes**	64.1%	64.1%	64.1%
- **No, but it's changing**	11.5%	10.3%	12.8%
**Contact your MP/councillor to express concerns about climate change**			
- **Yes**	73.1%	76.9%	69.2%
- **No, but it's changing**	7.1%	6.4%	7.7%
**Serve only vegan food at a party or social gathering**			
- **Yes**	28.8%	30.8%	26.9%
- **No, but it's changing**	19.9%	19.2%	20.5%
**Serve only vegetarian food at a party or social gathering**			
- **Yes**			
- **No, but it's changing**	41.0%	46.2%	35.9%
	15.4%	12.8%	17.9%

Supplementary Table 1.2 Social norms perceptions in the overall sample and in the two groups, control and treatment group.

As Table 1.2 shows the response profiles with respect to norms are usually quite similar across the two groups, with a couple of exceptions. In the control group a considerable higher number of people (still minority though) are considering taking a weekend flight for a European city break to go shopping or drinking as socially unacceptable, in the treatment group more respondent on the other hand perceived this norm as changing, towards becoming unacceptable. A z-test for proportions in two populations suggests that the difference with respect to not socially acceptable is insignificant ($z = 1.67$, $p = 0.095$), but the difference with respect to "yes, acceptable, but it is changing", is significant ($z = 2.69$, $p=0.007$). Whether this difference has any implications, will have to be investigated. Furthermore, a higher proportion of respondents from the treatment group perceived voting for a party, which plans to expand fossil fuel production, as socially unacceptable. But this difference was again not significant ($z = 1.13$, $p=0.258$). There are also some differences with respect to norms of serving vegan or vegetarian food, with more participants from the control group perceiving this as acceptable, but the differences are again insignificant (e.g. z-test for vegetarian food being acceptable $z = 1.30$, $p = 0.193$).

We conducted moreover a Multiple Correspondence Analysis (MCA) for the social norm variables, to see whether the responses can be summarised with fewer dimensions. We found that the anti-fossil fuel norms (working for fossil fuel company, investing in fossil fuels, voting for a party that would expand fossil fuel production) responses could be summarised rather well within a single dimension and created on that basis an index for anti-fossil fuel norms ranging from 3 to 9, with higher values indicating a stronger perception of established anti-fossil fuel norms. The other norms variables were more difficult to summarise in fewer dimensions (e.g. sufficiency dimension, political climate action dimension) as the MCAs suggested. We therefore left them as separate norm variables, turning them additionally into a set of dummy variables, measuring whether a given norm is perceived as established or emerging. Figure 1.10 shows the distribution for the anti-fossil fuel norm perception in the overall and the two groups, control and treatment group. The overall distribution and the distributions in both groups are skewed towards higher values, with central tendency towards higher values even more pronounced in the treatment group. However, a t-test suggests the difference in the means (6.88 in the control group and 7.27 in treatment group) is not significant (t = -1.27, p = 0.205).

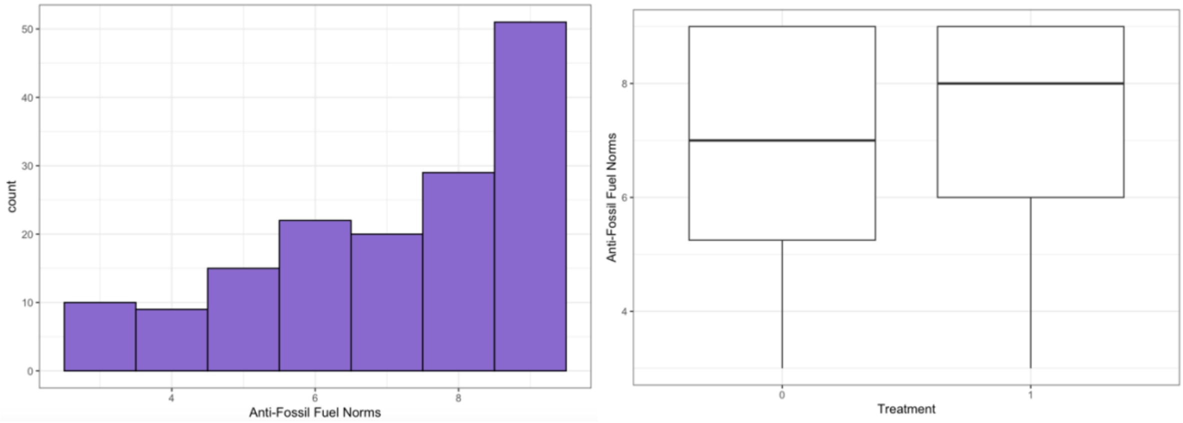

Supplementary Figure 1.10 Anti-fossil fuel social norms perception distribution in overall sample, and boxplots for the distribution in the two groups, control and experimental group.

Supplementary Note 1.6: Carbon Footprint Calibration Variables

Finally, we present here the data collected during user account set-up with the Climate Champ app on the **type of car** participants own, on the **type of heating system** they are using at their property and on their **average electricity usage**. Table 1.3 shows the relative frequencies of various car types in the overall sample and in the two groups, control and treatment group. Most of our participants own a standard petrol or diesel car. Electric cars are hardly represented in the study, slightly more in the control group than in the experimental group though. Quite a substantial number of people do not own a car at all, though they may still do car journeys, for instance using taxis or being driven by family members.

Car type	Total	Control Group	Treatment Group
**no car**	28.8%	30.8%	26.9%
**standard petrol/diesel**	55.8%	52.6%	59.0%
**electric**	6.4%	5.13%	3.8%
**SUV/4x4**	9.0%	9.0%	10.3%

Supplementary Table 1.3 Type of car owned in the overall sample and in the two groups, control and treatment group.

With respect to heating system (see Table 1.4), most of our participants use a modern gas boiler (i.e. not older than 5 years) to heat their homes. The second most common heating system is an old gas boiler, which produces higher CO_2 emissions. There is some difference with that respect between the two groups, there are more participants with modern gas boilers in the control group (z = 1.29, p = 0.197), while the treatment group has more participants with old gas boilers (z = 0.86, p = 0.390) and with electric heaters (z = 1.28, p = 0.201). Although these differences are not significant, it may still affect the carbon footprint of individual participants and the group average. Surprisingly few (just two participants) have an air source or ground source heat pump installed and those, who have

such a heating system are all in the control group (z = 1.42, p = 0.156). The no heating category indicate modern house builds (e.g. Passive House) that do not require heating.

Heating System	Total	Control Group	Treatment Group
**no heating**	1.3%	1.3%	1.3%
**electric**	10.9%	7.7%	14.1%
**air/ground heat pump**	1.3%	2.6%	0.0%
**modern gas boiler**	55.1%	60.3%	50.0%
**old gas boiler**	31.4%	28.2%	34.6%

Supplementary Table 1.4 Heating system used in property in the overall sample and in the two groups, control and treatment group.

The average electricity usage in the overall sample was 10.80 kWh (sd = 6.62). The means in the two groups are comparable (10.44 kWH in the control group, and 11.17 kWH in the treatment group), albeit slightly higher in the treatment group, though the difference is not significant (t = -0.69, p = 0.493).

Supplementary Note 1.7: Log-Transformation Overall Carbon Footprint

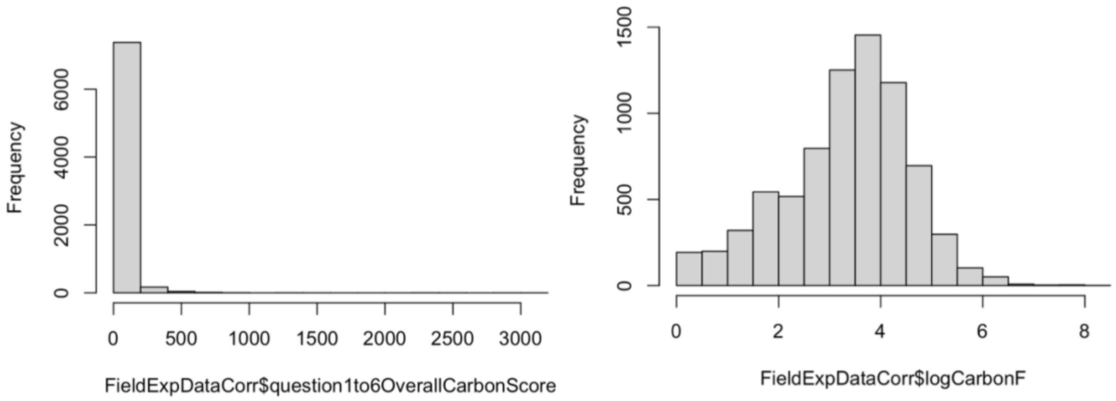

Supplementary Figure 1.11 Overall carbon footprint (left figure) with extreme skewness and log-transformed overall carbon footprint (right figure)

Finally, in Figure 1.11 we show why a log-transformation of the overall carbon footprint, calculated from the data collected through the app was necessary. The Figure clearly shows an extreme skewness towards lower values (around 10-20kgCO_2e), however there is a long tail of much higher values, due to outliers, resulting mostly from occasional flights, high-carbon purchases or long-distance car journeys.

Supplementary Note 2: Mixed Effect Models - Full Model Information

Here we test mainly the two pre-registered hypothesis 1 and 2 (see section 6), including testing hypothesis 1 also for the distinct domains heating, food, non-grocery consumption, electricity, taking flights and car journeys. As we also test the effect of time and its interaction with the treatment, we implicitly test also hypotheses 4 and 5 (see section 6).

Supplementary Note 2.1: Overall Carbon Footprint

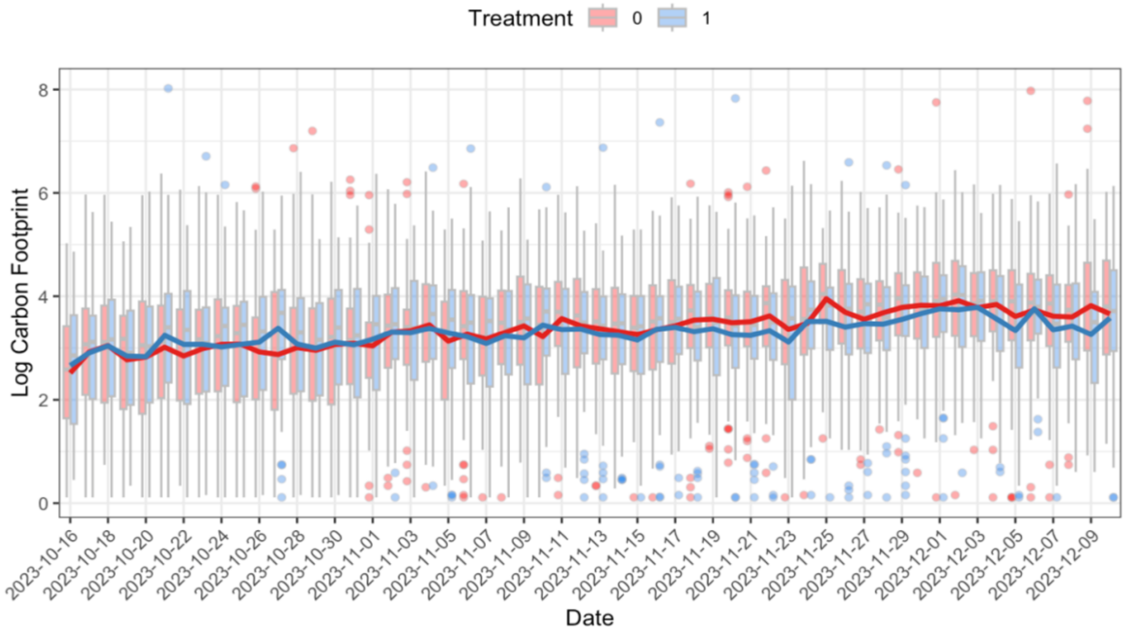

Supplementary Figure 2.1 Daily distributions of the log overall carbon footprint and the aggregate mean lines for the two groups

Model	AIC	BIC	LL	FE	LRT
M0 Base Model (Random Intercept)	21340	21340	-10667	-	-
M1 Mixed Effect Model (Random Intercept, Treatment Fixed Effect)	21342	21370	-10667	-0.033<br>(-0.29, 0.22)	0.062, p = 0.803
M2 Mixed Effect Model (Random Intercept, Treatment Random Slope, Treatment Fixed Effect)	21346	21387	-10667	-0.032<br>(-0.31, 0.23)	0.584, p = 0.9<br>0.522, p = 0.770
M3 Mixed Effect Model (Random Intercept, Time (Date) Fixed Effect)	20875	20903	-10434	0.0145<br>(0.01, 0.02)	467.22, p < 0.001
**M4 Mixed Effect Model (Random Intercept, with Interaction between Time (Date) and Treatment Fixed Effect)**	**20855**	**20897**	**-10422**	**-0.006**<br>**(-0.009, -0.004)**<br>**0.145**<br>**(-0.12, 0.42)**<br>**0.018**<br>**(0.016, 0.02)**	**490.83, p < 0.001**<br>**23.709, p < 0.001**
M5 Mixed Effect Model (Random Intercept, Treatment Randon Slope, with Interaction between Time (Date) and Treatment Fixed Effect)	20859	20914	-10422	-0.006<br>(-0.009, -0.004)<br>0.145<br>(-0.12, 0.43)<br>0.018<br>(0.016, 0.02)	491.27, p < 0.001<br>0.3385, p = 0.844

Supplementary Table 2.1 Model (M) outcome with Log Carbon Footprint as outcomes variable.
LL: Log Likelihood, FE: Fixed Effect with 95%-confidence interval in brackets, LRT: Likelihood Ratio Test. M1 is assessed against base model (M0). M2 is assessed against M0 and M1 (second line LRT). M3 is assessed against the based model (M0). M4 is assessed against M0 and M3 (second line LRT). M5 is assessed against M0 and M4. FE column for models (M4, M5) with interaction terms show first the interaction FE term, then the treatment FE and the time FE, with the respective confidence intervals in brackets underneath. Best model highlighted in bold.

Supplementary Note 2.2: Heating Carbon Footprint

Model	AIC	BIC	LL	FE	LRT
M0 Base Model (Random Intercept)	68987	69008	-34491	-	-
M1 Mixed Effect Model (Random Intercept, Treatment Fixed Effect)	68988	69015	-34490	-6.841<br>(-16.49, 3.16)	1.8343, p = 0.176
M2 Mixed Effect Model (Random Intercept, Treatment Random Slope, Treatment Fixed Effect)	68966	69008	-34477	-6.763<br>(-16.32, 3.34)	26.878, p < 0.001<br>25.043, p < 0.001
M3 Mixed Effect Model (Random Intercept, Treatment Random Slope, Treatment & Time (Date) Fixed Effect)	68278	68327	-34132	-6.919<br>(-17.14, 2.56)<br>0.395<br>(0.37, 0.43)	717.12, p < 0.001<br>690.24, p < 0.001
M4 Mixed Effect Model (Random Intercept, with Interaction between Time (Date) and Treatment Fixed Effect)	68290	68332	-34139	-0.098<br>(-0.16, -0.04)<br>-4.183<br>(-15.1, 6.19)<br>0.444<br>(0.41, 0.49)	703.09, p < 0.001<br>701.25, p < 0.001
**M5 Mixed Effect Model (Random Intercept, Treatment Random Slope, with Interaction between Time (Date) and Treatment Fixed Effect)**	**68269**	**68325**	**-34127**	**-0.098**<br>**(-0.15, -0.04)**<br>**-4.148**<br>**(-13.9, 6.77)**<br>**0.444**<br>**(0.40, 0.49)**	**728.19, p < 0.001**<br>**11.07, p = 0.001**<br>**25.11, p < 0.001**

Supplementary Table 2.2 Model (M) outcome with Heating Carbon Footprint as outcomes variable.
LL: Log Likelihood, FE: Fixed Effect with 95%-confidence interval in brackets, LRT: Likelihood Ratio Test. M1 is assessed against base model (M0). M2 is assessed against M0 and M1 (second line LRT). M3 is assessed against M0 and M2 (second line LRT). M4 is assessed against M0 and M1 (second line LRT). M5 is assessed against M0, M3 (second line LRT) and M4 (third line LRT). FE column for M3 shows first the treatment FE, then the time FE, with the respective confidence intervals in brackets underneath. FE column for models (M4, M5) with interaction terms show first the interaction FE term, then the treatment FE and the time FE, with the respective confidence intervals in brackets underneath. Best model highlighted in bold.

Supplementary Note 2.3: Food Carbon Footprint

Model	AIC	BIC	LL	FE	LRT
M0 Base Model (Random Intercept)	59044	59065	-29519	-	-
M1 Mixed Effect Model (Random Intercept, Treatment Fixed Effect)	59045	59073	-29519	0.697<br>(-1.29, 2.87)	0.4083, p = 0.523
M2 Mixed Effect Model (Random Intercept, Treatment Random Slope, Treatment Fixed Effect)	59024	59065	-29506	0.696<br>(-1.46, 2.93)	26.072, p < 0.001<br>25.663, p < 0.001
M3 Mixed Effect Model (Random Intercept, Treatment Random Slope, Treatment & Time (Date) Fixed Effect)	59023	59071	-29504	0.694<br>(-1.44, 2.72)<br>0.014<br>(-2e-03, 0.03)	28.951, p < 0.001<br>2.8796, p = 0.090
M4 Mixed Effect Model (Random Intercept, Treatment/Time (Date) Interaction Fixed Effect	59043	59085	-29516	-0.031<br>(-0.06, 0.002)<br>1.568<br>(-0.82, 3.79)<br>0.029<br>(0.01, 0.05)	6.6837, p = 0.083<br>6.2753, p = 0.043
**M5 Mixed Effect Model (Random Intercept, Treatment Random Slope, Treatment/Time (Date) Interaction Fixed Effect**	**59021**	**59077**	**-29503**	**-0.031<br>(-0.06, 0.004)<br>1.578<br>(-0.89, 3.82)<br>0.029<br>(0.006, 0.05)**	**32.568, p < 0.001<br>6.4963, p = 0.039<br>25.884, p < 0.001**

Supplementary Table 2.3 Model (M) outcome with Carbon Footprint from Food (dairy/meat) as outcomes variable. LL: Log Likelihood, FE: Fixed Effect with 95%-confidence interval in brackets, LRT: Likelihood Ratio Test. M1 is assessed against base model (M0). M2 is assessed against M0 and M1 (second line LRT). M3 is assessed against M0 and M2 (second line LRT). M4 is assessed against M0 and M1 (second line LRT). M5 is assessed against M0, M3 (second line LRT) and M4 (third line LRT). FE column for M3 shows first the treatment FE, then the time FE, with the respective confidence intervals in brackets underneath. FE column for models (M4, M5) with interaction terms show first the interaction FE term, then the treatment FE and the time FE, with the respective confidence intervals in brackets underneath. Best model highlighted in bold.

Supplementary Note 2.4: Non-Grocery Consumption Carbon Footprint

Model	AIC	BIC	LL	FE	LRT
M0 Base Model (Random Intercept)	72997	73018	-36495	-	-
M1 Mixed Effect Model (Random Intercept, Treatment Fixed Effect)	72998	73026	-36495	-1.004<br>(-3.16, 1.06)	0.916, p = 0.339
M2 Mixed Effect Model (Random Intercept, Treatment Random Slope, Treatment Fixed Effect)	72991	73033	-36490	-1.02<br>(-3.16, 0.88)	11.621, p = 0.009<br>0.705, p = 0.005
**M3 Mixed Effect Model (Random Intercept, Treatment Random Slope, Treatment & Time (Date) Fixed Effect)**	**72984**	**73032**	**-36485**	**-1.04**<br>**(-3.08, 1.07)**<br>**0.063**<br>**(0.02, 0.10)**	**20.9, p = 0.0003**<br>**9.28, p = 0.002**
M4 Mixed Effect Model (Random Intercept, Treatment/Time (Date) Interaction Fixed Effect	72991	73032	-36489	-0.06<br>(-0.14, 0.03)<br>0.594<br>(-2.52, 3.76)<br>0.092<br>(0.03, 0.15)	12.19, p = 0.007
M5 Mixed Effect Model (Random Intercept, Treatment Random Slope, Treatment/Time (Date) Interaction Fixed Effect	72984	73040	-36484	-0.06<br>(-0.13, 0.03)<br>0.555<br>(-2.61, 3.78)<br>0.091<br>(0.03, 0.15)	22.69, p = 0.0004<br>1.794, p = 0.1805<br>10.50, p = 0.005

Supplementary Table 2.4 Model (M) outcome with Carbon Footprint from Non-Grocery Consumption as outcome variable. LL: Log Likelihood, FE: Fixed Effect with 95%-confidence interval in brackets, LRT: Likelihood Ratio Test. M1 is assessed against base model (M0). M2 is assessed against M0 and M1 (second line LRT). M3 is assessed against M0 and M2 (second line LRT). M4 is assessed against M0. M5 is assessed against M0, M3 (second line LRT) and M4 (third line LRT). FE column for M3 shows first the treatment FE, then the time FE, with the respective confidence intervals in brackets underneath. FE column for models (M4, M5) with interaction terms show first the interaction FE term, then the treatment FE and the time FE, with the respective confidence intervals in brackets underneath. Best model highlighted in bold.

Supplementary Note 2.5: Electricity Carbon Footprint

Model	AIC	BIC	LL	FE	LRT
M0: Base Model (Random Intercept)	21352	21373	-10673	-	-
M1: Mixed Effect Model (Random Intercept, Treatment Fixed Effect)	21354	21381	-10673	-0.184<br>(-0.71, 0.32)	0.4769, p = 0.490
M2: Mixed Effect Model (Random Intercept, Treatment Random Slope, Treatment Fixed Effect)	21357	21399	-10672	-0.184<br>(-0.67, 0.38)	1.1091, p = 0.775<br>0.6321, p = 0.729
**M3: Mixed Effect Model (Random Intercept, Time (Date) Fixed Effect)**	**21234**	**21262**	**-10613**	**0.007**<br>**(0.006, 0.009)**	**120, p < 0.001**
M4: Mixed Effect Model (Random Intercept, Time (Date) and Treatment Fixed Effect)	21236	21270	-10613	-0.186<br>(-0.74, 0.33)<br>0.007<br>(0.006, 0.009)	120.49, p < 0,001<br>0.489, p = 0.484
M5: Mixed Effect Model (Random Intercept, Treatment/Time (Date) Interaction Fixed Effect	21237	21279	-10613	-0.001<br>(-0.004, 0.002)<br>-0.162<br>(-0.71, 0.32)<br>0.008<br>(0.006, 0.009)	120.87, p < 0.001<br>0.870, p = 0.647

Supplementary Table 2.5 Model (M) outcome with Electricity Carbon Footprint as outcomes variable
LL: Log Likelihood, FE: Fixed Effect with 95%-confidence interval in brackets, LRT: Likelihood Ratio Test. M1 is assessed against base model (M0). M2 is assessed against M0 and M1 (second line LRT). M3 is assessed against the M0. M4 is assessed against the M0 and M3 (second line LRT). M5 is assessed against M0 and M3. FE column for M4 shows first the treatment FE, then the time FE, with the respective confidence intervals in brackets underneath. FE column for M5 with interaction term shows first the interaction FE term, then the treatment FE and the time FE, with the respective confidence intervals in brackets underneath. Best model highlighted in bold.

Supplementary Note 2.6: Flying Carbon Footprint

Model	AIC	BIC	LL	FE	LRT
**M0: Base Model (Random Intercept)**	**88858**	**88879**	**-44426**	-	-
M1: Mixed Effect Model (Random Intercept, Treatment Fixed Effect)	88860	88888	-44426	-1.044<br>(-5.51, 3.54)	0.2058, p = 0.650
M2: Mixed Effect Model (Random Intercept, Treatment Random Slope, Treatment Fixed Effect)	88860	88901	-44424	-1.075<br>(-5.86, 3.33)	4.5437, p = 0.208<br>4.3379, p = 0.114
M3: Mixed Effect Model (Random Intercept, Time (Date) Fixed Effect)	88857	88885	-44425	0.100<br>(0.02, 0.21)	2.8561, p = 0.091
M4: Mixed Effect Model (Random Intercept, Treatment/Time (Date) Interaction Fixed Effect	88860	88901	-44424	-0.147<br>(-0.38, 0.07)<br>3.144<br>(-4.55, 11.44)<br>0.173<br>(0.02, 0.35)	4.617, p = 0.202

Supplementary Table 2.6 Model (M) outcome with Carbon Footprint from Flying as outcomes variable
LL: Log Likelihood, FE: Fixed Effect with 95%-confidence interval in brackets, LRT: Likelihood Ratio Test. Each of the models M1-M4 was assessed against the base model M0. Additionally, M2 was assessed against M1 (second line LRT). FE column for M4 with interaction term shows first the interaction FE term, then the treatment FE and the time FE, with the respective confidence intervals in brackets underneath. Best model highlighted in bold.

Supplementary Note 2.7: Car Journeys Carbon Footprint

Model	AIC	BIC	LL	FE	LRT
M0: Base Model (Random Intercept)	68152	68172	-34073	-	-
M1: Mixed Effect Model (Random Intercept, Treatment Fixed Effect)	68149	68177	-34071	4.557<br>(0.39, 9.01)	4.3495, p = 0.037
**M2: Mixed Effect Model (Random Intercept, Treatment Random Slope, Treatment Fixed Effect)**	**68089**	**68131**	**-34039**	**4.670<br>(0.55, 9.10)**	**68.484, p < 0.001<br>64.135, p < 0.001**
M3: Mixed Effect Model (Random Intercept, Treatment Random Slope, Treatment & Time (Date) Fixed Effect)	68091	68139	-34038	4.671<br>(0.33, 8.85)<br>-0.006<br>(-0.03, 0.02)	68.675, p < 0.001<br>0.1911, p = 0.662
M4: Mixed Effect Model (Random Intercept, Treatment Random Slope, with Treatment & Time (Date) Interaction Fixed Effect	68088	68144	-34036	-0.064<br>(-0.12, -0.01)<br>6.505<br>(1.72, 10.89)<br>0.026<br>(-0.02, 0.07)	73.40, p < 0.001<br>4.919, p = 0.09<br>4.727, p = 0.03

Supplementary Table 2.7 Model (M) outcome with Carbon Footprint from Car Journeys as outcomes variable LL: Log Likelihood, FE: Fixed Effect with 95%-confidence interval in brackets, LRT: Likelihood Ratio Test. M1 is assessed against base model (M0). M2 is assessed against M0 and M1 (second line LRT). M3 is assessed against M0 and M2. M4 is assessed against M0, M2 and M3. FE column for M3 shows first the treatment FE, then the time FE, with the respective confidence intervals in brackets underneath. FE column for M4 with interaction term shows first the interaction FE term, then the treatment FE and the time FE, with the respective confidence intervals in brackets underneath. Best model highlighted in bold.

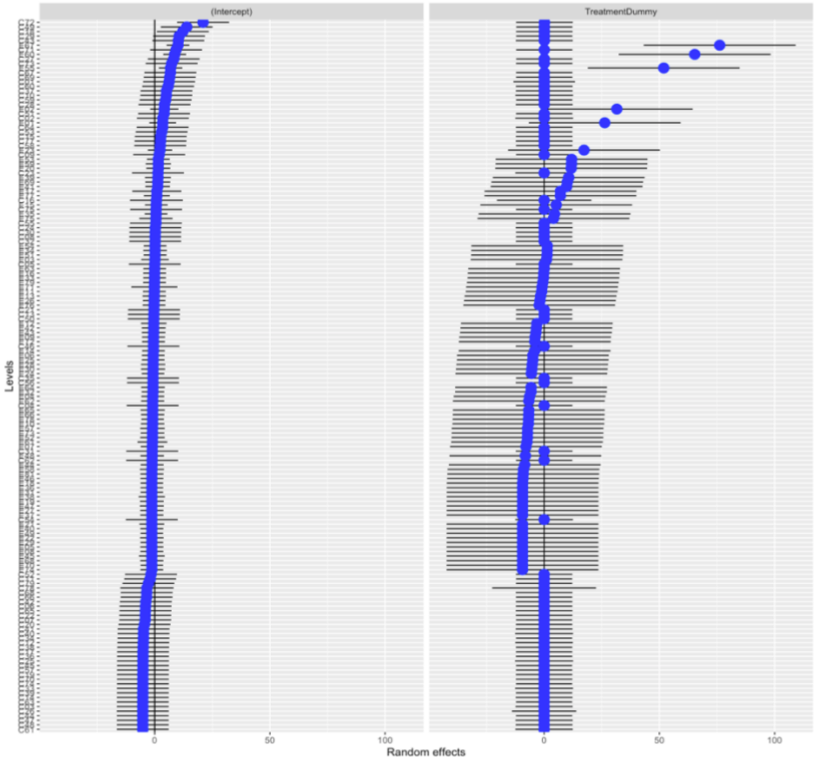

Supplementary Figure 2.2 Mixed Effects Model, Random Intercepts and Random Slope Visualisation. We can see the outliers with high positive slope estimates in the treatment group. The slope estimates around zero are mostly for control group study participants.

Supplementary Note 2.8: Civic Climate Positivity

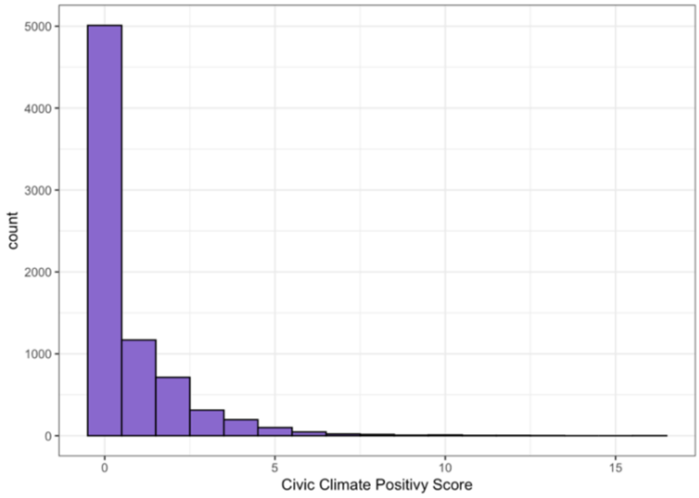

Supplementary Figure 2.3 shows the distribution for civic climate positivity score, a count variable of civic and political climate actions taken, across the sample. Given the excess of zeros, Zero-Inflated Poisson Mixed Effect Model will be estimated.

Model	AIC	BIC	LL	FE	LRT
M0 Base Model (Random Intercept)	15057	15072	-7523	-	-
M1 Zero-Inflated Poisson Mixed Effect Model (Random Intercept, Treatment Fixed Effect, Treatment Fixed Effect for Excess Zeros)	15054	15076	-7520	0.331<br>[0.07, 0.60]<br>(0.083)	6.36, p = 0.042
M2 Zero-Inflated Poisson Mixed Effect Model (Random Intercept, Treatment Fixed Effect, Time Fixed Effect for Excess Zeros)	15049	15071	-7518	0.334<br>[0.07, 0.59]<br>(-0.006)	11.29, p = 0.004
M3 Zero-Inflated Poisson Mixed Effect Model (Random Intercept, Treatment Random Slope, Treatment Fixed Effect, Time Fixed Effect for Excess Zeros)	15054	15084	-7517	0.317<br>[0.06, 0.58]<br>(-0.006)	12.64, p = 0.027<br>1.34, p = 0.719
**M4 Zero-Inflated Poisson Mixed Effect Model (Random Intercept, Treatment and Time Fixed Effect, Time Fixed Effect for Excess Zeros)**	**14994**	**15019**	**-7489**	**0.293**<br>**[0.04, 0.55]**<br>**0.008**<br>**[0.006, 0.01]**<br>**(0.003)**	**67.65, p < 0.001**<br>**56.36, p < 0.001**
M5 Zero-Inflated Poisson Mixed Effect Model (Random Intercept, with Treatment and Time Interaction Fixed Effect, Time Fixed Effect for Excess Zeros)	14995	15023	-7489	-0.002<br>[-0.006, 0.001]<br>0.362<br>[0.08, 0.65]<br>0.009<br>[0.006, 0.01]<br>(0.003)	69.36, p < 0.001<br>1.71, p = 0.191

Supplementary Table 2.8 Model outcome with Civic Climate Positivity as outcomes variable.
Note, differently from carbon footprint, here we want to increase the score. LL: Log Likelihood, FE: Fixed Effect with 95%-confidence interval in square brackets, LRT: Likelihood Ratio Test, Estimate of zero-part coefficient in round brackets. M1 is assessed against base model (M0). M2 is assessed against M0. M3 is assessed against M0 and M2. M4 is assessed against M0 and M2. M5 is assess against M0 and M4. FE column for M4 shows first the treatment FE, then the time FE, with the respective confidence intervals in square brackets underneath. FE column for M5 with interaction term shows first the interaction FE term, then the treatment FE and the time FE, with the respective confidence intervals in square brackets underneath. Best model highlighted in bold.

Supplementary Note 2.9: Models with Climate Positive Behaviour Index

Climate Positive Behaviour Index 1

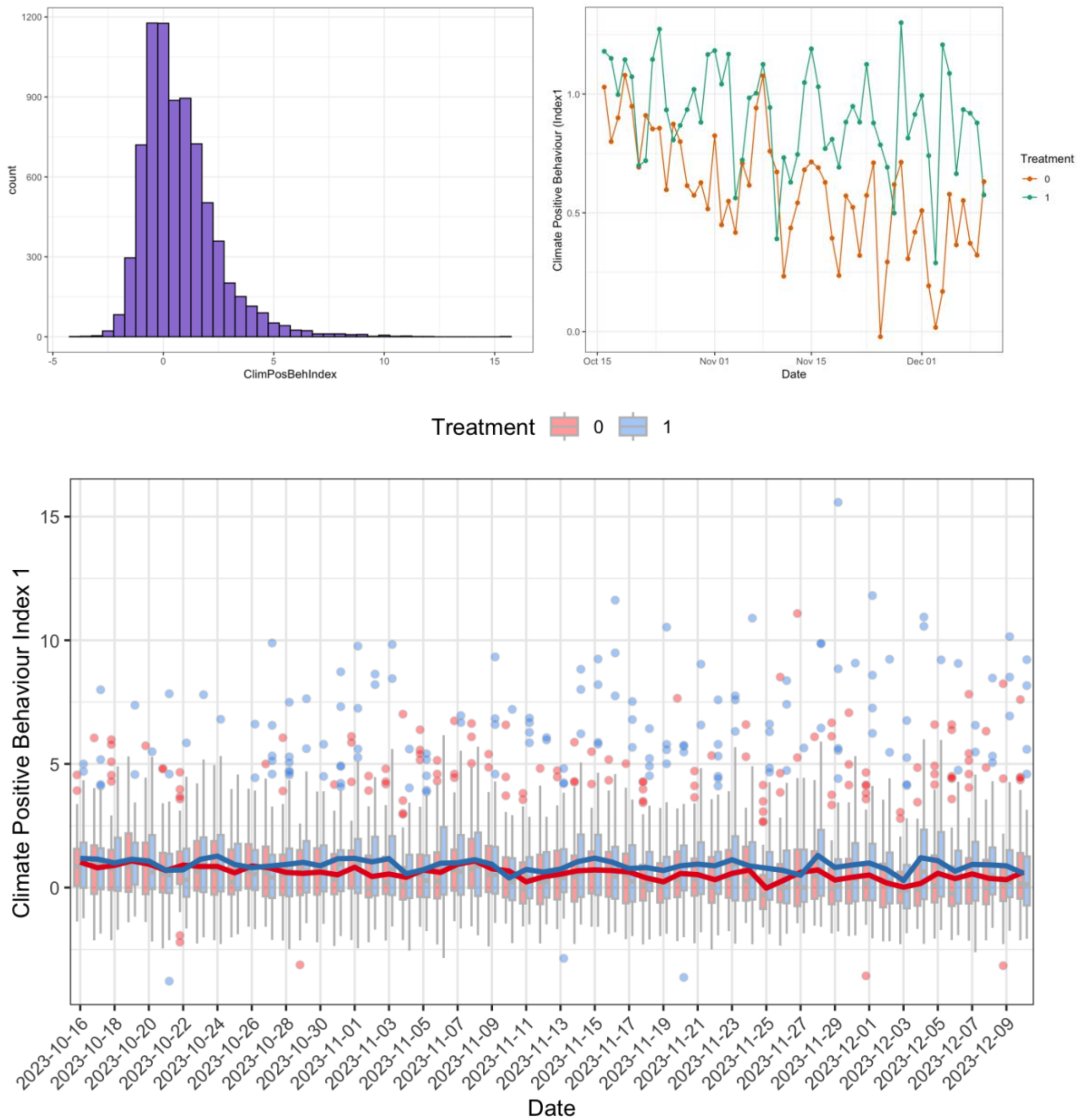

Supplementary Figure 2.4 shows the distribution for Climate Positive Behaviour Index 1, across the sample, aggregated mean lines for the two groups, treatment and control group and daily distributions of the Climate Positive Behaviour Index 1 along with the aggregate mean lines for the two groups

The third pre-registered hypothesis was formulated for an index combining the overall carbon footprint and civic climate positivity score into a single index. We created two combined indices. To create the first index, we centred the log carbon values, reversing their direction, so that negative values mean negative for climate change, as carbon footprint would be higher than average, and positive values would be positive for climate, as the carbon footprint would be lower than average. To this measure we then added the climate positivity score to create a single, combined outcome variable, climate positive behaviour. Figure 2.4 visualises the distribution for this index. Table 2.9 shows the models for this index, testing pre-registered hypothesis 3 (see section 6, and where time is included, also implicitly hypothesis 4, 5).

Model	AIC	BIC	LL	FE	LRT
M0 Base Model (Random Intercept)	26245	26266	-13120	-	-
M1 Mixed Effect Model (Random Intercept, Treatment Fixed Effect)	26245	26273	-13118	0.261<br>(-0.08, 0.64)	2.316, p = 0.128
M2 Mixed Effect Model (Random Intercept, Treatment Random Slope, Treatment Fixed Effect)	26245	26286	-13116	0.262<br>(-0.07, 0.58)	6.660, p = 0.084
M3 Mixed Effect Model (Random Intercept, Time (Date) Fixed Effect)	26204	26231	-13098	-0.006<br>(-0.008, -0.004)	43.83, p < 0.001
**M4 Mixed Effect Model (Random Intercept, with Interaction between Time (Date) and Treatment Fixed Effect)**	**26196**	**26237**	**-13092**	**0.006<br>(0.002, 0.01)<br>0.097<br>(-0.26, 0.43)<br>-0.009<br>(-0.01, -0.007)**	**55.81, p < 0.001<br>11.98, p = 0.003**
M5 Mixed Effect Model (Random Intercept, Treatment Randon Slope, with Interaction between Time (Date) and Treatment Fixed Effect)	26195	26251	-13090	0.006<br>(0.002, 0.009)<br>0.098<br>(-0.24, 0.45)<br>-0.009<br>(-0.012, -0.007)	60.29, p < 0.001<br>4.482, p = 0.106

Supplementary Table 2.9 Model (M) outcome with Climate Positive Behaviour Index 1 as outcomes variable. LL: Log Likelihood, FE: Fixed Effect with 95%-confidence interval in brackets, LRT: Likelihood Ratio Test. M1 is assessed against base model (M0). M2 is assessed against M0 and M1 (second line LRT). M3 is assessed against the based model (M0). M4 is assessed against M0 and M3 (second line LRT). M5 is assessed against M0 and M4. FE column for models (M4, M5) with interaction terms show first the interaction FE term, then the treatment FE and the time FE, with the respective confidence intervals in brackets underneath. Best model highlighted in bold. See Figure 2.6 for visualisation of interaction effect.

Climate Positive Behaviour Index 2

The second index was created based on distance from an ideal carbon daily carbon footprint as a benchmark, namely 6.8 kg CO2, which is the maximum permitted value per person in 2030 if we would want to ensure we stay within the 1.5 Degrees climate warming threshold (see Akenji et al. 2021). To this distance measure, which again has positive values if one stays below the benchmark and negative values if one has higher carbon footprint, we added again the civic climate positivity score. Figure 2.5 visualises the distribution for this index. Table 2.10 shows the models for this index, testing pre-registered hypothesis 3 (see section 6, and where time is included, also implicitly hypothesis 4, 5).

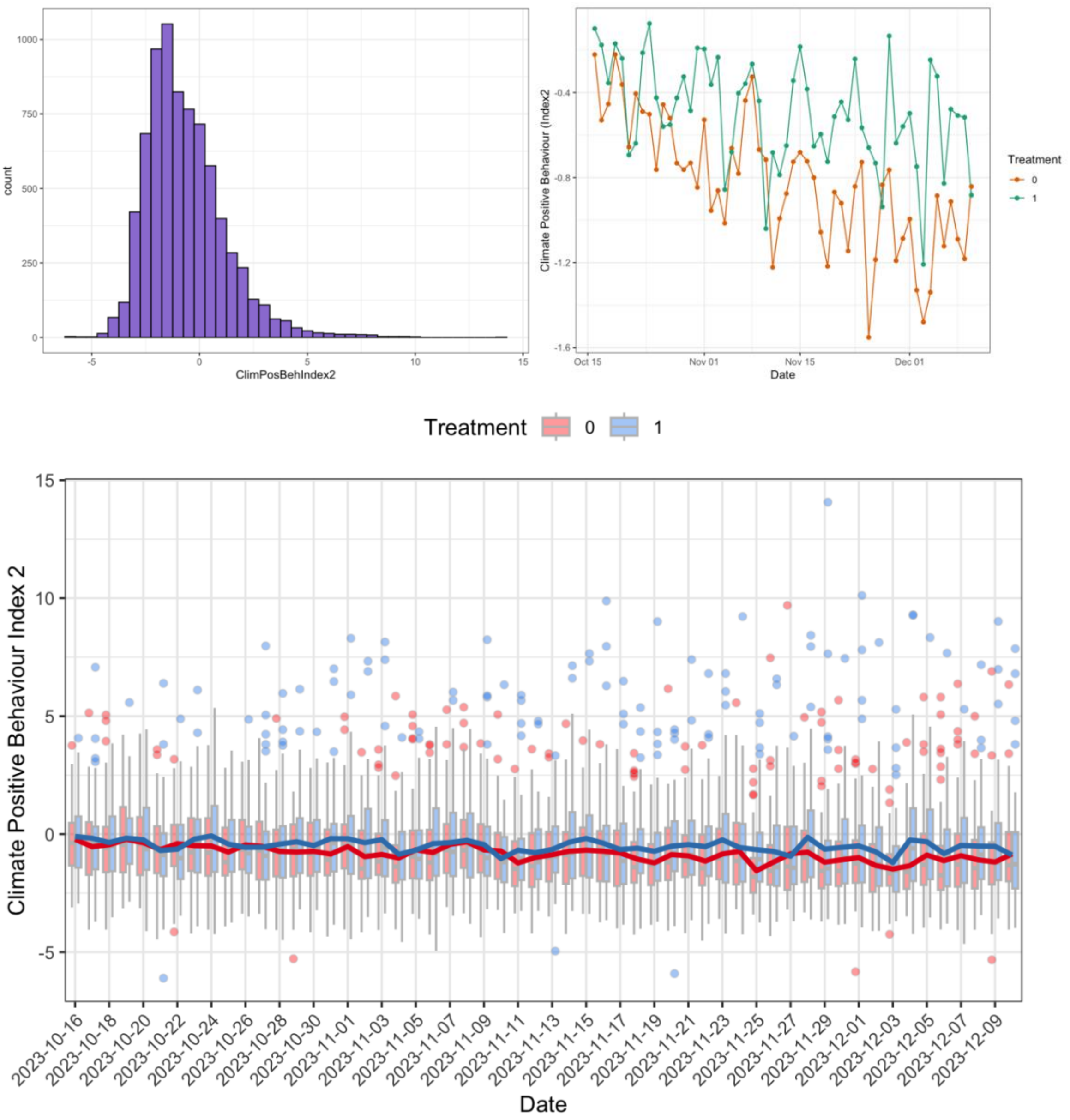

Supplementary Figure 2.5 shows the distribution for Climate Positive Behaviour Index 2, across the sample, aggregated mean lines for the two groups, treatment and control group and daily distributions of the Climate Positive Behaviour Index 2 along with the aggregate mean lines for the two groups

Model	AIC	BIC	LL	FE	LRT
M0 Base Model (Random Intercept)	27453	27474	-13723	-	-
M1 Mixed Effect Model (Random Intercept, Treatment Fixed Effect)	27453	27480	-13722	0.267<br>(-0.12, 0.64)	2.042, p = 0.153
M2 Mixed Effect Model (Random Intercept, Treatment Random Slope, Treatment Fixed Effect)	27455	27496	-13721	0.269<br>(-0.10, 0.61)	4.202, p = 0.240
M3 Mixed Effect Model (Random Intercept, Time (Date) Fixed Effect)	27376	27403	-13684	-0.009<br>(-0.01, -0.007)	79.09, p < 0.001
**M4 Mixed Effect Model (Random Intercept, with Interaction between Time (Date) and Treatment Fixed Effect)**	**26196**	**26237**	**-13092**	**0.007<br>(0.003, 0.01)<br>0.069<br>(-0.34, 0.46)<br>-0.013<br>(-0.02, -0.009)**	**93.35, p < 0.001<br>14.26, p < 0.001**
M5 Mixed Effect Model (Random Intercept, Treatment Randon Slope, with Interaction between Time (Date) and Treatment Fixed Effect)	26195	26251	-13090	0.007<br>(0.003, 0.01)<br>0.070<br>(-0.29, 0.46)<br>-0.013<br>(-0.015, -0.01)	95.65, p < 0.001<br>2.299, p = 0.317

Supplementary Table 2.10 Model (M) outcome with Climate Positive Behaviour Index 2 as outcomes variable. LL: Log Likelihood, FE: Fixed Effect with 95%-confidence interval in brackets, LRT: Likelihood Ratio Test. M1 is assessed against base model (M0). M2 is assessed against M0 and M1 (second line LRT). M3 is assessed against the based model (M0). M4 is assessed against M0 and M3 (second line LRT). M5 is assessed against M0 and M4. FE column for models (M4, M5) with interaction terms show first the interaction FE term, then the treatment FE and the time FE, with the respective confidence intervals in brackets underneath. Best model highlighted in bold. See Figure 2.6 for visualisation of interaction effect.

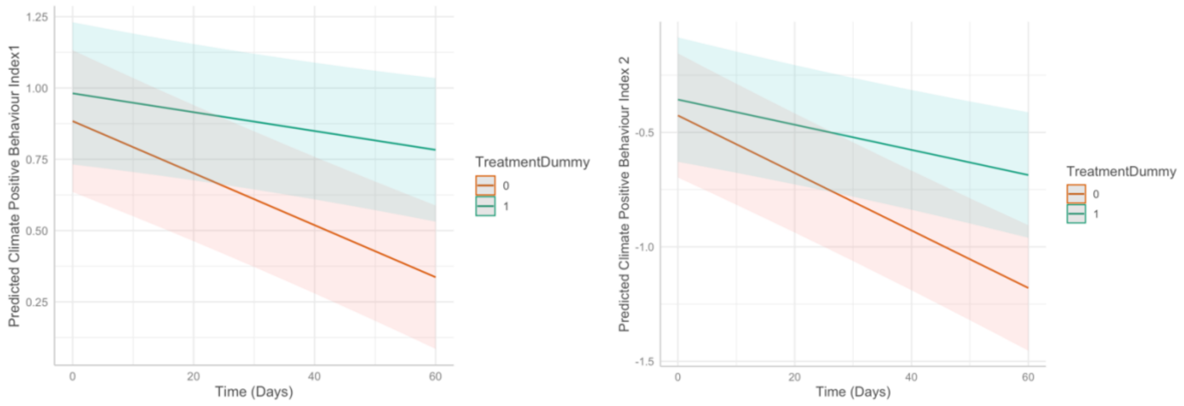

Supplementary Figure 2.6 shows the interaction effect for the respective Model 4 for the two indices, i.e. effect of the interaction between treatment and time on Climate Positive Behaviour 1 and 2.

Supplementary Note 3: Covariate Models

Supplementary Note 3.1: Political leaning

Model	AIC	BIC	LL	FE (Covar.)	LRT
M4 for Log Carbon Footprint overall (Table SI2.1) + political leaning FE	20856	20904	-10421	0.04<br>(-0.02, 0.10)	1.653, p = 0.199
**M4 for Civic Climate Positivity Score (Table SI2.8) + political leaning and treatment interaction FE**	**14993**	**15023**	**-7486**	**0.118<br>(-0.001, 0.24)<br>-0.187<br>(-0.80, 0.42)<br>-0.094<br>(-0.18, -0.01)<br>0.008<br>(0.006, 0.01)**	**6.35, p = 0.042**
M5 for Heating Carbon Footprint (Table SI2.2) + political leaning FE	68270	68332	-34126	-1.305<br>(-3.28, 0.67)	1.610, p = 0.205
**M5 for Food Carbon Footprint (Table SI2.3) + political leaning FE**	**59015**	**59077**	**-29498**	**0.632<br>(0.20, 1.08)**	**8.342, p = 0.004**
**M2 for Car Usage Carbon Footprint (Table SI2.7) + political leaning FE**	**68084**	**68133**	**-34035**	**0.913<br>(0.28, 1.59)**	**7.085, p = 0.008**
M3 for Non-Grocery Consumption Carbon Footprint (Table SI2.4) + political leaning FE	72984	73040	-36484	0.281<br>(-0.21, 0.76)	1.391, p = 0.238
M3 for Electricity Carbon Footprint (Table SI2.5) political leaning FE	21236	21271	-10613	0.018<br>(-0.10, 0.15)	0.082, p = 0.774
M0 for Flying Footprint (Table SI2.6) + political leaning FE	88859	88886	-44425	-0.680<br>(-1.70, 0.38)	1.510, p = 0.219

Supplementary Table 3.1 Best respective models from SI2 with left-right political self-placement fixed effect (FE) added, reported are fixed effects and 95% CI for the covariate. The model with added left-right self-placement fixed effect is assessed against the respective best model via Likelihood Radio Test (LRT). FE column for model for civic climate positive score with interaction term shown first, then the treatment FE, then the political leaning FE and then the FE for time, with the respective confidence intervals in brackets underneath. Models with significant covariate fixed effect in bold.

Please note, we have also explored models with interaction between treatment and left-right self-placement, but since none of these models produced any significant interaction effects, with exception for the civic climate positivity score, which is included in Table 3.1, they are not further reported here (they are included in the R Markdown file that includes all covariate analyses).

Supplementary Note 3.2: Values

Altruistic Values

Model	AIC	BIC	LL	FE (Covar.)	LRT
M4 for Log Carbon Footprint overall (Table 2.1) + altruistic values FE	20857	20906	-10422	0.01 (-0.13, 0.15)	0.017, p = 0.897
M4 for Civic Climate Positivity (Table 2.8) + altruistic values FE	14996	15024	-7489	-0.002 (-0.15, 0.14)	0.740, p = 0.389
M5 for Heating Carbon Footprint (Table 2.2) + altruistic values FE	68271	68333	-34126	1.634 (-3.51, 6.73)	0.431, p = 0.512
M5 for Food Carbon Footprint (Table 2.3) + altruistic values FE	59022	59085	-29502	-0.556 (-1.65, 0.51)	1.089, p = 0.297
M2 for Car Usage Carbon Footprint (Table 2.7) + altruistic values FE	68091	68139	-34038	-0.534 (-2.17, 1.15)	0.404, p = 0.525
M3 for Non-Grocery Consumption Carbon Footprint (Table 2.4) + altruistic values FE	72986	73041	-36485	0.301 (-0.87, 1.59)	0.261, p = 0.610
M3 for Electricity Carbon Footprint (Table 2.5) + altruistic values FE	21235	21269	-10612	0.181 (-0.11, 0.46)	1.406, p = 0.236
M0 for Flying Footprint (Table 2.6) + altruistic values FE	88859	88887	-44426	-1.352 (-4.09, 1.19)	1.004, p = 0.316

Supplementary Table 3.2 Best respective models from section 2 with altruistic values fixed effect (FE) added, reported are fixed effects and 95% CI for the covariate. The model with added altruistic values fixed effect is assessed against the respective best model via Likelihood Radio Test (LRT). Models with significant covariate fixed effect in bold (none in this case).

Please note, we have also explored models with interaction between treatment and altruistic values, but since none of these models produced any significant interaction effects, they are not further reported here (they are included in the R Markdown file that includes all covariate analyses).

Biospheric Values

Model	AIC	BIC	LL	FE (Covar.)	LRT
M4 for Log Carbon Footprint overall (Table 2.1) + biospheric values FE	20857	20905	-10421	0.051 (-0.09, 0.18)	0.553, p = 0.457
M4 for Civic Climate Positivity (Table 2.8) + biospheric values FE	14997	15024	-7490	-0.001 (-0.14, 0.13)	0.31, p = 0.5788
**M5 for Heating Carbon Footprint (Table 2.2) + biospheric values FE**	**68267**	**68329**	**-34124**	**4.866 (-0.08, 9.78)**	**4.321, p = 0.038**
M5 for Food Carbon Footprint (Table 2.3) + biospheric values FE	59021	59084	-29502	-0.635 (-1.56, 2.01)	1.936, p = 0.164
M2 for Car Usage Carbon Footprint (Table 2.7) + biospheric values FE	68089	68137	-34037	-1.122 (-2.46, 0.28)	2.588, p = 0.108
M3 for Non-Grocery Consumption Carbon Footprint (Table 2.4) + biospheric values FE	72985	73040	-36484	0.605 (-3.48, 1.69)	1.290, p = 0.256
M3 for Electricity Carbon Footprint (Table 2.5) + biospheric values FE	21236	21271	-10613	0.063 (-0.21, 0.32)	0.204, p = 0.652
**M0 for Flying Footprint (Table 2.6) + biospheric values FE**	**88856**	**88883**	**-44424**	**-2.524 (-4.93, -0.20)**	**4.465, p = 0.035**

Supplementary Table 3.3 Best respective models from section 2 with biospheric values fixed effect (FE) added, reported are fixed effects and 95% CI for the covariate. The model with added biospheric values fixed effect is assessed against the respective best model via Likelihood Radio Test (LRT). Models with significant covariate fixed effect in bold.

Please note, we have also explored models with interaction between treatment and biospheric values, but since none of these models produced any significant interaction effects, they are not further reported here (they are included in the R Markdown file that includes all covariate analyses). The significant results for biospheric values are not included in the main paper, as they seem to be overall less significant in contributing to our understanding. Although it appears that higher levels of biospheric values are correlated with higher carbon footprint from heating, the effect is somewhat in doubt, as the confidence interval includes negative and positive values. More pronounced is the negative relation between high levels of biospheric values and carbon footprint from taking flights, however, as data on flights is sparse, this relation too should be treated with some caution.

Egoistic Values

Model	AIC	BIC	LL	FE (Covar.)	LRT
M4 for Log Carbon Footprint overall (Table 2.1) + egoistic values FE	20857	20906	-10422	0.018 (-0.11, 0.15)	0.079, p = 0.779
**M4 for Civic Climate Positivity (Table 2.8) + egoistic values and treatment interaction FE**	**14991**	**15022**	**-7586**	**0.362 (0.11, 0.62) -0.855 (-1.73, 0.02) -0.232 (-0.43, -0.04) 0.008 (0.006.0.01)**	**7.43, p = 0.024**
**M5 for Heating Carbon Footprint (Table 2.2) + egoistic values FE**	**68266**	**68328**	**-34124**	**-4.680 (-8.78, -0.78)**	**5.33, p = 0.021**
**M3 for Food Carbon Footprint (Table 2.3) + egoistic values and treatment interaction FE**	**59018**	**59081**	**-29500**	**2.333 (0.38, 4.41) -7.300 (-14.3, -0.50) 0.218 (-0.72, 1.27) 0.014 (-0.002, 0.03)**	**5.057, p = 0.024**
**M2 for Car Usage Carbon Footprint (Table 2.7) + egoistic values and treatment interaction FE**	**68087**	**68142**	**-34035**	**5.066 (0.89, 9.50) -12.557 (-27.70, 1.76) -0.673 (-2.23, 0.87)**	**6.108, p = 0.040**
M1 for Non-Grocery Consumption Carbon Footprint (Table 2.4) + egoistic values FE	72984	73040	-36484	0.622 (-0.30, 1.54)	1.713, p = 0.191
M3 for Electricity Carbon Footprint (Table 2.5) + egoistic values FE	21235	21269	-10612	0.155 (-0.1, 0.39)	1.454, p = 0.228
M0 for Flying Footprint (Table 2.6) + egoistic values FE	88860	88888	-44426	-0.313 (-2.51, 2.05)	0.077, p = 0.781

Supplementary Table 3.4 Best respective models from section 2 with egoistic values fixed effect (FE) added, reported are fixed effects and 95% CI for the covariate. The model with egoistic fixed effect is assessed against the respective best model via Likelihood Radio Test (LRT). FE column for models for civic climate positive score, food carbon footprint and car usage carbon footprint with interaction term show first the interaction FE term, then the treatment FE, then the political leaning FE and then the FE for time (except for car usage carbon footprint model, where time was insignificant), with the respective confidence intervals in brackets underneath. Models with significant covariate fixed effect in bold.

Egoistic values were more prevalent among male participants (M=3.43) comparing to female or trans and non-binary participants (M=3.24) (t =-8.004, p<0.001). They are also more prevalent amongst people who identify as politically right (r =0.223, p<0.001).

Hedonic Values

Model	AIC	BIC	LL	FE (Covar.)	LRT
M4 for Log Carbon Footprint overall (Table 2.1) + hedonic values FE	20857	20906	-10422	-0.041 (-0.20, 0.12)	0.251, p = 0.617
M4 for Civic Climate Positivity (Table 2.8) + hedonic values FE	14994	15022	-7488	-0.107 (-0.25, 0.04)	2.66, p = 0.103
M5 for Heating Carbon Footprint (Table 2.2) + hedonic values FE	68270	68332	-34126	-3.174 (-8.92, 2.51)	1.272, p = 0.260
M5 for Food Carbon Footprint (Table 2.3) + hedonic values FE	59023	59086	-29503	-0.026 (-1.07, 1.05)	0.002, p = 0.961
M2 for Car Usage Carbon Footprint (Table 2.7) + hedonic values FE	68091	68140	-34039	0.238 (-1.36, 1.89)	0.080, p = 0.778
M3 for Non-Grocery Consumption Carbon Footprint (Table 2.4) + hedonic values FE	72986	73041	-36485	0.243 (-1.01, 1.50)	0.147, p = 0.702
M3 for Electricity Carbon Footprint (Table 2.5) + hedonic values FE	21235	21270	-10612	-0.174 (-5.20, 1.71)	1.103, p = 0.294
M0 for Flying Footprint (Table 2.6) + hedonic values FE	88860	88888	-44426	-0.290 (-3.27, 2.52)	0.040, p = 0.841

Supplementary Table 3.5 Best respective models from section 2 with hedonic values fixed effect (FE) added, reported are fixed effects and 95% CI for the covariate. The model with added hedonic values fixed effect is assessed against the respective best model via Likelihood Radio Test (LRT). Models with significant covariate fixed effect in bold (none in this case).

Please note, we have also explored models with interaction between treatment and hedonic values, but since none of these models produced any significant interaction effects, they are not further reported here (they are included in the R Markdown file that includes all covariate analyses).

Supplementary Note 3.3: Social Norms Perceptions

Model	AIC	BIC	LL	FE (Covar.)	LRT
M4 for Log Carbon Footprint overall (Table 2.1) + anti-fossil fuel norms index FE	20855	20903	-10420	-0.057 (-0.12, 0.01)	2.669, p = 0.102
M4 for Civic Climate Positivity (Table 2.8) + anti-fossil fuel norms index FE	14997	15025	-7490	-0.016 (-0.09, 0.06)	0.37, p = 0.541
M5 for Heating Carbon Footprint (Table 2.2)	Not tested, as no specific social norm with respect to heating assessed				
M5 for Food Carbon Footprint (Table 2.3) + vegetarian/vegan diet social norms FE	Tested were eight models with established and dynamic vegetarian and vegan social norm (serving vegan/vegetarian food only at party acceptable) and models with interaction term with treatment, no model was of better fit than M5				
M2 for Car Usage Carbon Footprint (Table 2.7) + driving social norm FE	Tested were four models with established and dynamic driving social norm (driving walkable distance acceptability) and models with interaction term with treatment, no model was of better fit than M2				
M3 for Non-Grocery Consumption Carbon Footprint (Table 2.4) + pro-consumer lifestyle social norm FE	Tested were four models with established and dynamic pro-consumer lifestyle social norm (city break flying to go shopping/drinking acceptable) and models with interaction term with treatment, no model was of better fit than M3				
M3 for Electricity Carbon Footprint (Table 2.5) + energy social norm FE	Tested were four models with established and dynamic energy social norm (leaving lights on acceptability) and models with interaction term with treatment, no model was of better fit than M3				
M0 for Flying Footprint (Table 2.6) + flying social norm FE	Tested were four models with established and dynamic flying social norm (flying when train connection available acceptability) and models with interaction term with treatment, no model was of better fit than M0				

Supplementary Table 3.6 Models with social norms (summary), please see R Markdown file for all model details

We did not find any effect from social norms. We note however, that we did not try to manipulate social norms or their perception, which has been found to be affective in changing behaviour in previous studies. Without changing social norms or their perception, social norms are unlikely to explain variation in individual behaviour, as social norms are widely shared. We still find some evidence that social norms can affect behaviour. Specifically, when testing a logistic regression model with voting intention for a party with ambitious climate policy as the binary outcome variable, the established social norm around non-acceptability of voting for a party that wants to expand fossil fuel production had a significant effect (0.89 [95%-CI: 0.21, 1.58], p = 0.01). On the other hand, perceiving this social norm as changing (dynamic social norm) did not have a significant effect.

We note moreover that although we did not find any significant effect from anti-fossil fuel norms on the summary indices Log Carbon Footprint and Civic Climate Positivity Score. We found that the treatment changed participant's anti-fossil fuel norms perception. While in participants from both groups anti-fossil fuel norms were more prevalent after participating in the study (Initial Mean: 7.08, Post-Study Mean: 7.4, Diff =-0.34 [95%-CI: -0.60, -0.08], t=-2.59, p=0.01), this was only significant in the case of the experimental group (Initial Mean: 7.27, Post-Study Mean: 7.65, Diff =-0.41 [95%-CI: -0.75, -0.07], t=-2.43, p=0.017) compared to the control group (Initial Mean: 6.89, Post-Study Mean: 7.15, Diff =-0.27 [95%-CI: -0.67, 0.13], t=-1.33, p=0.189). Which is another indication for the effectiveness of our intervention in revealing the moral implications of fossil fuels.

Supplementary Note 3.4: Climate Change Worry

Model	AIC	BIC	LL	FE (Covar.)	LRT
M4 for Log Carbon Footprint overall (Table 2.1) + climate change worry FE	20857	20906	-10422	0.016 (-0.19, 0.21)	0.026, p = 0.872
M4 for Civic Climate Positivity (Table 2.8) + climate change worry FE	14995	15023	-7489	0.102 (-0.1, 0.30)	1.57, p = 0.211
M5 for Heating Carbon Footprint (Table 2.2) + climate change worry FE	68268	68331	-34125	5.573 (-1.42, 11.95)	2.656, p = 0.103
M5 for Food Carbon Footprint (Table 2.3) + climate change worry FE	59020	59083	-29501	-1.106 (-2.38, 1.80)	2.812, p = 0.094
M2 for Car Usage Carbon Footprint (Table 2.7) + climate change worry FE	68091	68140	-34039	0.084 (-1.96, 2.23)	0.007, p = 0.932
M3 for Non-Grocery Consumption Carbon Footprint (Table 2.4) climate change worry FE	72985	73041	-36485	-0.653 (-2.19, 0.82)	0.725, p = 0.394
M3 for Electricity Carbon Footprint (Table 2.5) + climate change worry FE	21236	21270	-10613	0.151 (-0.28, 0.57)	0.565, p = 0.452
M0 for Flying Footprint (Table 2.6) + climate change worry FE	88858	88886	-44425	-2.585 (-6.21, 0.91)	2.233, p = 0.135

Supplementary Table 3.7 Best respective models from section 2 with climate change worry fixed effect (FE) added, reported are fixed effects and 95% CI for the covariate. The model with added climate change worry fixed effect is assessed against the respective best model via Likelihood Radio Test (LRT). Models with significant covariate fixed effect in bold (none in this case).

Please note, we have also explored models with interaction between treatment and climate change concern, but since none of these models produced any significant interaction effects, they are not further reported here (they are included in the R Markdown file that includes all covariate analyses).

In accordance with hypothesis 11 (see section 6) we also tested whether the treatment and participation in the study affected people's climate change concerns, and we found no significant change (treatment group: t=-1.73, p=0.09, control group: t=-0.44, p=0.67), in climate change concerns when comparing concerns before and after study participation. Hence, we reject hypothesis 11.

Supplementary Note 3.5: Panel Models with time-varying covariate sense of agency and time-in varying socio-demographic covariates

Sense of agency with respect to climate change was measured through the app Climate Champ daily, it is therefore a time-varying covariate and required therefore panel regression approach. We can take first a look at the average trends of the sense of agency in the two groups, treatment and control group (Figure 3.1). It seems the two groups are very similar in their sense of agency. There is also no clear trend, the average trend appears more like a wave. Throughout the study respondents were more likely to respond that they did not feel agency (5991 out of 7615 responses, 78.7%) rather than that they felt agency (1624 out of 7615 responses, 21.3%). It does not seem, at least at the aggregate level, that the sense of agency improved e.g. through the app providing information about possible actions (e.g. contacting MP). Though one may want to explore trends at the individual level. Reported below are not only the within and between effect of sense of agency (testing hypothesis 13, see SI6), but also the effects of various socio-demographic variables (testing hypotheses 17 (income) and 18 (gender), see SI6, but, exploring also possible other covariates).

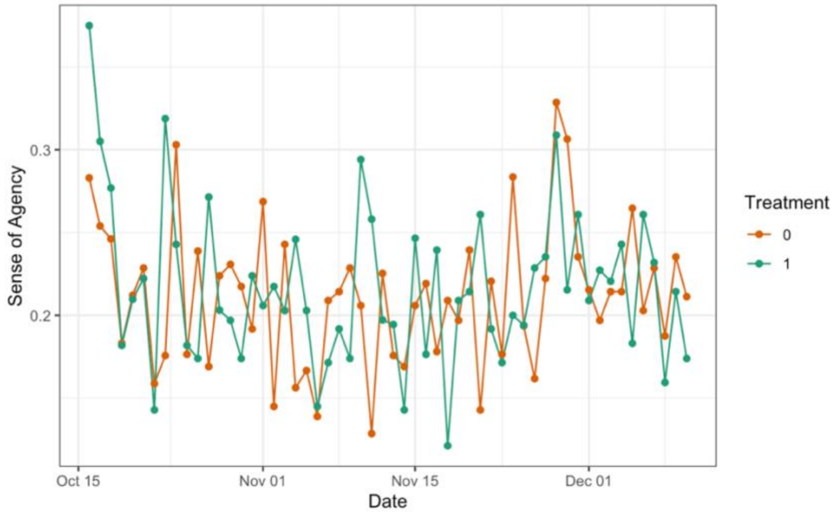

Supplementary Figure 3.1 Aggregated mean line of sense of agency in the two groups treatment and control group.

Log Carbon Footprint

Variable	Est	95%CI	p-value
Within Effects:			
**Sense of agency**	**0.11**	**0.04, 0.18**	**< 0.001**
Between Effects:			
Sense of agency	0.07	-0.42, 0.55	0.80
**Income**	**0.13**	**0.03, 0.23**	**0.01**
Rural (dummy)	-0.07	-0.40, 0.27	0.70
Age	0.01	-0.004, 0.02	0.25
Male (dummy)	0.00	-0.26, 0.26	1.00
Homeowner (dummy)	0.10	-0.17, 0.38	0.47
Academic education (dummy)	-0.08	-0.36, 0.20	0.59
**Having children (dummy)**	**0.30**	**0.03, 0.57**	**0.04**
White British (dummy)	0.17	-0.11, 0.45	0.25

Supplementary Table 3.8 Panel Regression (within-between model) for log carbon footprint with sense of agency and a set of socio-demographic covariates as predictors. AIC = 21363.65, BIC = 21453.84, Pseudo-R^2 (fixed effects) = 0.05, Pseudo-R^2 (total) = 0.44, Entity ICC = 0.41. Significant effects in bold.

Civic Climate Positivity Score

Variable	Est	95%CI	p-value
Within Effects:			
**Sense of agency**	**0.67**	**0.56, 0.78**	**< 0.001**
Between Effects:			
**Sense of agency**	**1.31**	**0.85, 1.76**	**< 0.001**
**Income**	**-0.11**	**-0.21, -0.02**	**0.02**
**Rural (dummy)**	**0.58**	**0.27, 0.90**	**< 0.001**
Age	-0.01	-0.02, 0.005	0.31
Male (dummy)	0.06	-0.19, 0.30	0.61
Homeowner (dummy)	0.09	-0.18, 0.36	0.33
Academic education (dummy)	0.22	-0.01, 0.53	0.12
**Having children (dummy)**	**0.30**	**0.04, 0.56**	**0.03**
**White British (dummy)**	**-0.31**	**-0.57, -0.04**	**0.02**
**Treatment**	**0.29**	**0.04, 0.53**	**0.02**
Cross-Level Interactions:			
**Sense of agency * Treatment**	**0.21**	**0.06, 0.36**	**0.01**

Supplementary Table 3.9 Panel Regression (within-between model) for civic climate positivity score with sense of agency and a set of socio-demographic covariates as predictors. This model includes also a significant cross-level interaction between treatment and sense of agency. AIC = 23243.66co, BIC = 23347.73, Pseudo-R^2 (fixed effects) = 0.16, Pseudo-R^2 (total) = 0.43, Entity ICC = 0.32. Significant effects in bold.

Car Journeys Carbon Footprint

Variable	Est	95%CI	p-value
Within Effects:			
Sense of agency	0.73	-0.73, 2.19	0.33
Between Effects:			
Sense of agency	-3.67	-11.63, 4.32	0.38
Income	0.78	-0.82, 2.39	0.36
Rural (dummy)	2.78	-2.71, 8.32	0.34
Age	0.03	-0.16, 0.23	0.75
Male (dummy)	4.43	0.16, 8.68	0.05
Homeowner (dummy)	0.27	-4.29, 4.83	0.91
Academic education (dummy)	-3.15	-7.81, 1.48	0.20
**Having children (dummy)**	**7.00**	**2.49, 11.48**	**<0.01**
White British (dummy)	-0.09	-4.70, 4.54	0.97

Supplementary Table 3.10 Panel Regression (within-between model) for car journeys carbon footprint with sense of agency and a set of socio-demographic covariates as predictors. AIC = 68121.24, BIC = 68211.43, Pseudo-R^2 (fixed effects) = 0.04, Pseudo-R^2 (total) = 0.31, Entity ICC = 0.28. Significant effects in bold.

Food Carbon Footprint

Variable	Est	95%CI	p-value
Within Effects:			
Sense of agency	0.25	-0.56, 1.05	0.55
Between Effects:			
Sense of agency	2.84	-1.13, 6.80	0.17
Income	0.28	-0.52, 1.07	0.51
Rural (dummy)	2.32	-0.42, 5.07	0.11
Age	-0.08	-0.18, 0.02	0.13
Male (dummy)	1.05	-1.07, 3.17	0.34
Homeowner (dummy)	1.90	-0.38, 4.17	0.11
Academic education (dummy)	-1.39	-3.70, 0.92	0.25
Having children (dummy)	1.28	-0.94, 3.52	0.28
**White British (dummy)**	**-3.06**	**-5.36, -0.77**	**0.01**

Supplementary Table 3.11 Panel Regression (within-between model) for food carbon footprint with sense of agency and a set of socio-demographic covariates as predictors. AIC = 59029.81, BIC = 59120, Pseudo-R^2 (fixed effects) = 0.03, Pseudo-R^2 (total) = 0.26, Entity ICC = 0.24. Significant effects in bold.

Heating Carbon Footprint

Variable	Est	95%CI	p-value
Within Effects:			
**Sense of agency**	**3.37**	**1.86, 4.89**	**<0.01**
Between Effects:			
Sense of agency	0.08	-18.70, 18.84	0.99
**Income**	**4.19**	**0.44, 7.94**	**0.03**
Rural (dummy)	-10.10	-22.94, 2.75	0.14
**Age**	**0.66**	**0.19, 1.12**	**0.01**
Male (dummy)	-1.12	-11.14, 8.90	0.83
Homeowner (dummy)	-0.12	-10.79, 10.52	0.98
Academic education (dummy)	-0.58	-11.46, 10.31	0.92
Having children (dummy)	-3.25	-13.80, 7.30	0.56
**White British (dummy)**	**8.80**	**-2.03, 19.64**	**0.12**

Supplementary Table 3.12 Panel Regression (within-between model) for heating carbon footprint with sense of agency and a set of socio-demographic covariates as predictors. AIC = 59029.81, BIC = 59120, Pseudo-R^2 (fixed effects) = 0.03, Pseudo-R^2 (total) = 0.26, Entity ICC = 0.24. Significant effects in bold.

Electricity Carbon Footprint

Variable	Est	95%CI	p-value
Within Effects:			
**Sense of agency**	**0.09**	**0.03, 0.16**	**<0.01**
Between Effects:			
Sense of agency	-0.06	-1.01, 0.89	0.90
Income	0.09	-0.10, 0.28	0.37
**Rural (dummy)**	**0.87**	**0.22, 1.52**	**0.01**
Age	-0.01	-0.03, 0.01	0.47
**Male (dummy)**	**-0.59**	**-1.10, -0.08**	**0.03**
Homeowner (dummy)	0.46	-0.08, 1.00	0.11
Academic education (dummy)	0.15	-0.40, 0.70	0.61
**Having children (dummy)**	**0.80**	**0.27, 1.34**	**<0.01**
White British (dummy)	-0.05	-0.60, 0.50	0.87

Supplementary Table 3.13 Panel Regression (within-between model) for electricity carbon footprint with sense of agency and a set of socio-demographic covariates as predictors. AIC = 21358.4, BIC = 21448.6, Pseudo-R^2 (fixed effects) = 0.12, Pseudo-R^2 (total) = 0.77, Entity ICC = 0.74. Significant effects in bold.

Non-Grocery Consumption Carbon Footprint

Variable	Est	95%CI	p-value
	Within Effects:		
Sense of agency	1.42	-0.63, 3.46	0.17
	Between Effects:		
Sense of agency	3.63	-0.23, 7.48	0.07
Income	-0.62	-1.41, 0.16	0.13
Rural (dummy)	1.46	-1.27, 4.19	0.31
**Age**	**-0.11**	**-0.20, -0.01**	**0.04**
Male (dummy)	-1.17	-3.23, 0.91	0.28
Homeowner (dummy)	0.76	-1.48, 2.99	0.52
Academic education (dummy)	-1.58	-3.85, 0.69	0.19
Having children (dummy)	1.84	-0.34, 4.03	0.11
White British (dummy)	-0.06	-2.31, 2.20	0.96

Supplementary Table 3.14 Panel Regression (within-between model) for non-grocery consumption carbon footprint with sense of agency and a set of socio-demographic covariates as predictors. AIC = 72985.81, BIC = 73076.01, Pseudo-R^2 (fixed effects) = 0, Pseudo-R^2 (total) = 0.03, Entity ICC = 0.03. Significant effects in bold.

Flying Carbon Footprint

Variable	Est	95%CI	p-value
	Within Effects:		
Sense of agency	3.55	-2.28, 9.38	0.23
	Between Effects:		
Sense of agency	0.40	-8.02, 8.79	0.93
Income	1.15	-0.57, 2.87	0.21
Rural (dummy)	-3.33	-9.35, 2.66	0.29
Age	0.08	-0.13, 0.29	0.47
Male (dummy)	-3.92	-8.45, 0.61	0.10
**Homeowner (dummy)**	**6.24**	**1.33, 11.13**	**0.02**
Academic education (dummy)	1.71	-3.26, 6.68	0.51
Having children (dummy)	-2.82	-7.61, 1.96	0.26
White British (dummy)	-2.50	-7.45, 2.43	0.34

Supplementary Table 3.14 Panel Regression (within-between model) for carbon footprint from taking flights, with sense of agency and a set of socio-demographic covariates as predictors. AIC = 88830.06, BIC = 88920.25, Pseudo-R^2 (fixed effects) = 0, Pseudo-R^2 (total) = 0.01, Entity ICC = 0.01. Significant effects in bold.

The sense of agency effect for log carbon footprint at the within level is unclear and it is even less clear how to interpret it given the results for specific carbon footprint domains, where sense of agency did not play a role (apart for heating and electricity again at the within level). We hence assume that overall sense of agency did not play much of a role with respect to carbon footprint and the within effects are likely due to some confounders.

We tested cross-level interactions for all outcomes, but only for the civic climate positivity score there was a significant cross-level interaction with treatment. We also tested some interaction models with socio-demographics. Specifically, we investigate the interaction of the male dummy variable with treatment for car journeys and food carbon footprint (see hypothesis 18, see section 6), but did not find any significant interaction effect. With respect to hypothesis 17 (see section 6), we can see that income influences the overall carbon footprint, as predicted, those more affluent have a higher carbon footprint.

Supplementary Note 3.6: Panel Models with time-varying covariate emotional state

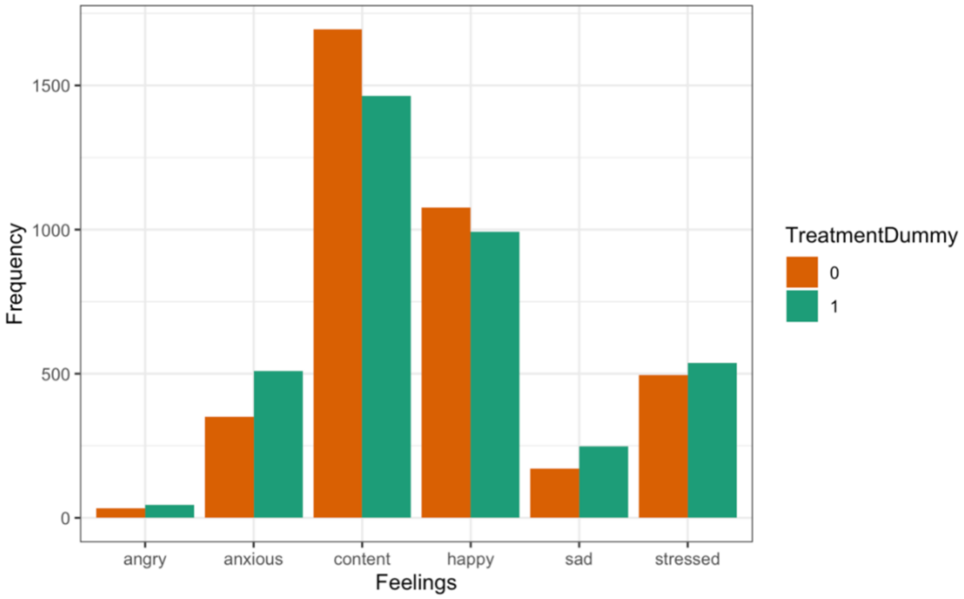

Supplementary Figure 3.2 Prevalence of various emotions in the two groups across the study.

If we look at the emotions, which participants expressed throughout the study on a daily basis, we can see that indeed a majority of the times, it was a positive emotion, content (3157) or happy (2068), which is together 5225 out of 7615, or 68.6%. With respect to negative emotions, the most common one was feeling stressed (1034 out of 7615, or 13.6%) and then feeling anxious (860 out of 7615, or 11.3%). The other two negative emotions, sad and angry were less common and in particular feeling angry was extremely rare. When we compare the emotional responses in the two groups, we can see some differences. Participants in the treatment group were more likely to report negative emotions and less likely to report positive emotions. A Chi-Square test (X^2=67.85, p<0.001) confirms that these differences are not random, but rather of a systematic nature. It appears, the exposure to the moral messages had some emotive consequences. Given this, we need to explore the effect that emotions have on our main outcome variables. We created a negative emotions dummy, combining “sad”, “stressed” and “anxious” for subsequent modelling purposes. We also tested the hypothesis 14 (see section 6) in this context.

Log Carbon Footprint

Variable	Est	95%CI	p-value
Within Effects:			
**Negative emotions (dummy)**	**-0.08**	**-0.14, -0.03**	**< 0.001**
Between Effects:			
Negative emotions (dummy)	-0.55	-0.42, 0.55	0.05
**Income**	**0.12**	**0.03, 0.23**	**0.02**
Rural (dummy)	-0.06	-0.40, 0.27	0.72
Age	0.01	-0.004, 0.02	0.24
Male (dummy)	-0.03	-0.26, 0.26	0.80
Homeowner (dummy)	0.07	-0.17, 0.38	0.63
Academic education (dummy)	-0.05	-0.36, 0.20	0.74
**Having children (dummy)**	**0.30**	**0.03, 0.57**	**0.04**
White British (dummy)	0.12	-0.11, 0.45	0.43

Supplementary Table 3.15 Panel Regression (within-between model) for overall log carbon footprint, with negative emotions dummy and a set of socio-demographic covariates as predictors. AIC = 21361.52, BIC = 21451.71, Pseudo-R^2 (fixed effects) = 0.06, Pseudo-R^2 (total) = 0.44, Entity ICC = 0.4. Significant effects in bold.

Variable	Est	95%CI	p-value
Within Effects:			
**Negative emotions (dummy)**	**-0.08**	**-0.13, -0.02**	**0.01**
**Sense of agency**	**0.11**	**0.04, 0.17**	**< 0.001**
Sense of agency * Negative emotions (dummy)	0.09	-0.08, 0.26	0.30
Between Effects:			
Negative emotions (dummy)	-0.56	-1.09, -0.03	0.05
Sense of agency	0.11	-0.36, 0.59	0.65
**Income**	**0.12**	**0.03, 0.22**	**0.02**
Rural (dummy)	-0.06	-0.39, 0.26	0.72
Age	0.01	-0.004, 0.02	0.24
Male (dummy)	-0.04	-0.29, 0.22	0.80
Homeowner (dummy)	0.06	-0.21, 0.33	0.63
Academic education (dummy)	-0.05	-0.32, 0.23	0.74
**Having children (dummy)**	**0.31**	**0.04, 0.57**	**0.04**
White British (dummy)	0.11	-0.17, 0.39	0.43

Supplementary Table 3.16 Panel Regression (within-between model) for overall log carbon footprint, with negative emotions dummy, sense of agency and interaction between them, as well as a set of socio-demographic covariates as predictors. AIC = 21361.82, BIC = 21465.89, Pseudo-R^2 (fixed effects) = 0.06, Pseudo-R^2 (total) = 0.44, Entity ICC = 0.4. Significant effects in bold.

To check whether sense of agency and emotional state could potentially be confounding factors, we also run a model, including both, which suggests they are not. We also tested potentially cross-level

interaction between treatment and negative emotions, but did not find any significant interaction (see R Markdown file for all tests). Furthermore, to test hypothesis 14 (see section 6), we also run a model where negative emotions and sense of agency interact, but this interaction was not significant (see Table 3.16).

We tested also the effect of negative emotions on carbon footprint in various domains. We can summarise, negative emotions had no effect whether at within or between level on car journeys carbon footprint or on food carbon footprint or on heating carbon footprint, or on electricity carbon footprint or on flying carbon footprint (see R Markdown file). They only affected negatively non-grocery consumption carbon footprint (i.e. negative emotions would lower it) at the within level (see Table 3.17).

Non-Grocery Consumption Carbon Footprint

Variable	Est	95%CI	p-value
Within Effects:			
**Negative emotions (dummy)**	**-2.29**	**-3.92, -0.66**	**0.01**
Between Effects:			
Negative emotions (dummy)	-3.71	-8.08, 0.65	0.11
Income	-0.70	-1.49, 0.09	0.09
Rural (dummy)	1.59	-1.14, 4.33	0.27
**Age**	**-0.11**	**-0.20, -0.01**	**0.04**
Male (dummy)	-1.35	-3.44, 0.74	0.22
Homeowner (dummy)	0.59	-1.68, 2.84	0.62
Academic education (dummy)	-1.25	-3.53, 1.03	0.30
Having children (dummy)	2.04	-0.14, 4.24	0.08
White British (dummy)	-0.27	-2.57, 2.02	0.82

Supplementary Table 3.17 Panel Regression (within-between model) for non-grocery consumption carbon footprint, with negative emotions dummy and a set of socio-demographic covariates as predictors. AIC = 72980.87, BIC = 73071.06, Pseudo-R^2 (fixed effects) = 0.01, Pseudo-R^2 (total) = 0.03, Entity ICC = 0.03. Significant effects in bold.

Civic Climate Positivity Score

Variable	Est	95%CI	p-value
Within Effects:			
**Negative emotions (dummy)**	**-0.18**	**-0.24, -0.12**	**< 0.001**
Between Effects:			
Negative emotions (dummy)	0.09	-0.48, 0.66	0.77
**Income**	**-0.12**	**-0.23, -0.02**	**0.03**
**Rural (dummy)**	**0.55**	**0.20, 0.91**	**< 0.001**
Age	-0.01	-0.02, 0.005	0.26
Male (dummy)	0.08	-0.19, 0.36	0.57
Homeowner (dummy)	0.18	-0.12, 0.47	0.25
Academic education (dummy)	0.27	-0.03, 0.56	0.09
**Having children (dummy)**	**0.33**	**0.04, 0.62**	**0.03**
White British (dummy)	-0.23	-0.52, 0.07	0.15
**Treatment**	**0.29**	**0.04, 0.53**	**0.02**

Supplementary Table 3.18 Panel Regression (within-between model) for civic climate positivity score, with negative emotions dummy and a set of socio-demographic covariates as predictors. AIC = 23628.86, BIC = 23719.05, Pseudo-R^2 (fixed effects) = 0.06, Pseudo-R^2 (total) = 0.4, Entity ICC = 0.36. Significant effects in bold.

Variable	Est	95%CI	p-value
Within Effects:			
**Negative emotions (dummy)**	**-0.14**	**-0.20, -0.08**	**< 0.001**
**Sense of agency**	**0.76**	**0.68, 0.84**	**< 0.001**
Sense of agency * Negative emotions (dummy)	-0.09	-0.29, 0.10	0.34
Between Effects:			
Negative emotions (dummy)	-0.05	-0.58, 0.47	0.84
Sense of agency	1.31	0.84, 1.78	< 0.001
**Income**	**-0.11**	**-0.21, -0.02**	**0.02**
**Rural (dummy)**	**0.54**	**0.22, 0.86**	**< 0.001**
Age	-0.01	-0.02, 0.005	0.31
Male (dummy)	0.06	-0.19, 0.31	0.64
Homeowner (dummy)	0.13	-0.14, 0.40	0.35
Academic education (dummy)	0.22	-0.05, 0.50	0.12
**Having children (dummy)**	**0.30**	**0.04, 0.57**	**0.03**
**White British (dummy)**	**-0.31**	**-0.59, -0.03**	**0.03**

Supplementary Table 3.19 Panel Regression (within-between model) for civic climate positivity score, with negative emotions dummy, sense of agency and interaction between them, as well as a set of socio-demographic covariates as predictors. AIC = 23239.89, BIC = 23350.89, Pseudo-R^2 (fixed effects) = 0.15, Pseudo-R^2 (total) = 0.43, Entity ICC = 0.33. Significant effects in bold.

Supplementary Note 4: Spillover Effect

To explore spillover effects, we first computed one-lag differences for each of our domain-specific carbon footprint outputs and for the civic and political climate action output. We then used bivariate panel regressions to estimate correlations between these output variables. We also estimated the same models using lagged difference (one lag) as predictors. The results are shown below in a correlation matrix (Table 4.1), which reports the panel regression within-effect coefficients.

	Heating CF Diff.	Food CF Diff.	Car CF Diff.	Consume CF Diff.	Electricity CF Diff.	Flying CF Diff.	Civic/Political CA Diff.
Heating CF Diff.		0.02	**-0.03*** **	0.01	**4.55*** **	**-0.01*** **	0.50*
Food CF Diff.	0.01		0.00	0.01*	0.24	-0.00	0.28*
Car CF Diff.	**-0.05*** **	0.01		0.00	0.07	0.01*	0.08
Consume CF Diff.	0.04	0.07*	0.01		0.48	0.00	**1.21*** **
Electricity CF Diff.	**0.01*** **	0.00	0.00	0.00		-0.001*	**0.04*** **
Flying CF Diff.	**-0.21*** **	-0.08	0.12*	0.00	-2.57*		0.23
Civic/Political CA Diff.	0.001*	0.001*	0.00	**0.001*** **	**0.08*** **	0.00	

Supplementary Table 4.1 CF: carbon footprint, Diff: one-lag difference, CA: climate action, *** p < 0.001, ** p < 0.01, * p < 0.05. Numbers are within effect coefficients for column variable being predictor!

There is little evidence for positive (i.e. promoting climate positive behaviour) spillovers effects at daily level, beyond for heating and electricity carbon footprint, i.e. people who try to reduce their carbon footprint from heating on a given day, would also lower their energy demand on the same day, reducing carbon footprint from electricity. There is also a positive, but very weak spillover effect between reducing carbon footprint from food and from non-grocery consumption. Another positive spillover effect is suggested between car journeys and flying, though that may just be due to the fact that people would often drive to the airport, hence higher carbon footprint from car journey on a given day would be correlated with higher carbon footprint from flying the same day. We have otherwise results for possible negative correlations, which may or may not showcase compensation or moral licensing behaviour (negative spillover effects). For instance, people who have decreased their carbon footprint from heating on a given day, tend to have higher carbon footprint from car journeys on the same day, which could signify moral licensing behaviour. The same can be said for the relation between heating and flying carbon footprint and electricity and flying carbon footprint, though that may again just be a correlation, because when people fly on a given day, they would automatically use less electricity and heating at home that day. More difficult to interpret are the spillover effects between civic and political climate action and carbon footprint in domains such as non-grocery consumption, electricity and to a lesser extent food and heating. Given we want to decrease carbon footprint but increase climate action the positive coefficient suggests that on a day when people have been engaged more in civic and political climate action, they tend to have greater carbon footprint from consumption, electricity, food and heating, though the effects are really rather small. This could hint to compensation behaviour, i.e. people felt somewhat guilty for having had a bigger carbon footprint on a given day and tried to compensate this by engaging at least in civic and political climate action. Any effect on behaviour (carbon footprint reduction) that spills over from civic and political climate action is likely to be lagged and not immediate. For that reason, we also looked at lagged effects (Table 4.2).

Indeed if we look at lagged effects from civic and political climate action on carbon footprint, we can see some spillover effects, where civic and political climate action lead to reduced carbon footprint at least in some domains (electricity, consumption and food though here the effect needs longer (lag of four) to take effect, on the other hand efforts to reduce carbon footprint can spill over with a lagged effect into greater civic and political climate action, most of the effects are however rather weak. We have also a positive, but weak spillover effect with lagged effect from car journey carbon footprint to heating carbon footprint, specifically participants, who would have tried to reduce their car journeys carbon footprint, may later try to reduce also their carbon footprint from heating, which is contrary to what we found without lagging the effect. Other effects we found without including lagged effects

disappeared or reversed, e.g. for heating and electricity, which may suggest moral licensing, not least if we also take into account the lagged autocorrelations. It seems generally, if people tried to reduce their carbon footprint in some domain, they revert to increased carbon footprint the next day, though it works also the other way round, if people had higher carbon footprint in a domain one day, they would try to reduce it the next day. Indeed, if we look at individual level data (see section 5), we can see typical day to day fluctuations, though over longer terms trends can emerge, albeit at the individual level these two can be rather non-linear.

	Heating CF Diff.	Food CF Diff.	Car CF Diff.	Consume CF Diff.	Electricity CF Diff.	Flying CF Diff.	Civic/Political CA Diff.
Heating CF Diff.	**-0.40*** **	0.00	0.02*	-0.01	**-2.21*** **	0.01	-0.20
Food CF Diff.	0.00	**-0.46*** **	0.01	-0.01	-0.02	0.00	-0.22<br>**-0.50*** (L4)**
Car CF Diff.	0.02	0.02	**-0.44*** **	-0.01	-0.29	0.00	-0.32
Consume CF Diff.	-0.02	-0.03	0.00	**-0.50*** **	-0.25	0.00	-0.83*
Electricity CF Diff.	**-0.001*** **	0.00	0.00	0.00	**-0.44*** **	0.00	**-0.03*** **
Flying CF Diff.	0.04	0.13	0.05	-0.03	0.08	**-0.47*** **	-0.08
Civic/Political CA Diff.	0.00	-0.001*	0.00	-0.001*	-0.03	0.00	**-0.49*** **

Supplementary Table 4.2 CF: carbon footprint, Diff: one-lag difference, CA: climate action, *** p < 0.001, ** p < 0.01, * p < 0.05. Numbers are within effect coefficients for lagged (1-lag) column variable being predictor! For difference in car journeys carbon footprint and in food carbon footprint we also tested lags up to five of civic and political climate action. One-lag auto-correlation in grey cells.

Supplementary Note 5: Some selected individual trajectories of change

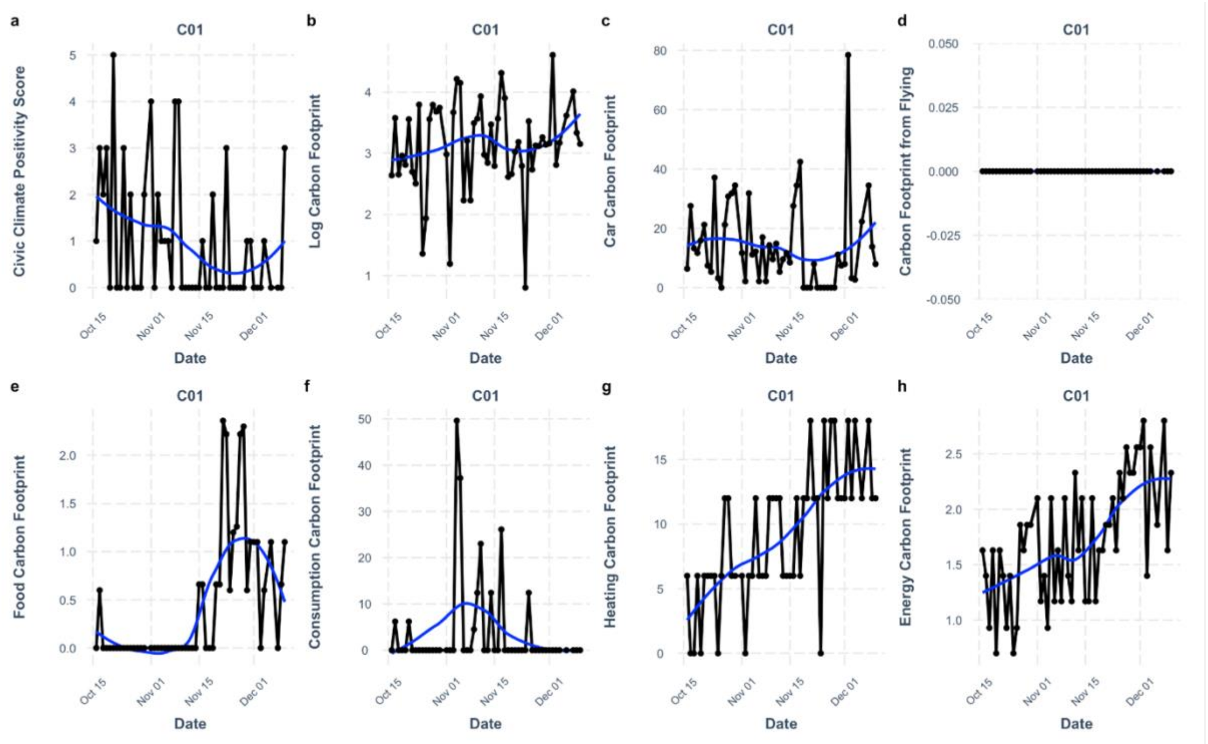

Supplementary Figure 5.1 Dynamic behavioural profile for study participant CO1 (control group)

The data offers also interesting insights into the behavioural dynamics at the individual level of each study participant, and we will research that data in greater detail and hope other researchers will do so too. Here we just present some snapshots to highlight the potential of the data and that individual change trajectories are rarely straightforward and linear, but our fine-grained data can provide insights into what drives those non-linear dynamics at the individual level.

Figure 5.1 for instance presents the behavioural dynamics for participant CO1 from the control group, who took part in the study from the start and until the end. What is noticeable is that at the start of the study they engaged more in civil/political activities around climate change, but this declined over the course of the study, with an increase again at the end. Their log carbon footprint went up, then down, then up again. A similar pattern can be seen for carbon footprint from driving. They did not fly in the study period. Their food carbon footprint was low to start with, after an initial higher score, but then increased again at the second half of the study, since mid-November. Their consumption carbon footprint went up and down again. The carbon footprint from heating increased predictably and so did their energy usage carbon footprint. From their open-text comments we could categorise the participant as low-key engaged, who mention particularly low-key efforts such as recycling in their comments, indeed they seemed to be mostly concerned about waste rather than GHG emissions. Their motivation is rather low, they wrote for instance one day: I could have read more and taken more action, but I haven't felt up too much today." or on a different day: "I had the heating on longer than probably needed." Still, they reported some efforts, like opting for buying second-hand clothes or singing a petition once. They also reported seeing climate disinformation online.

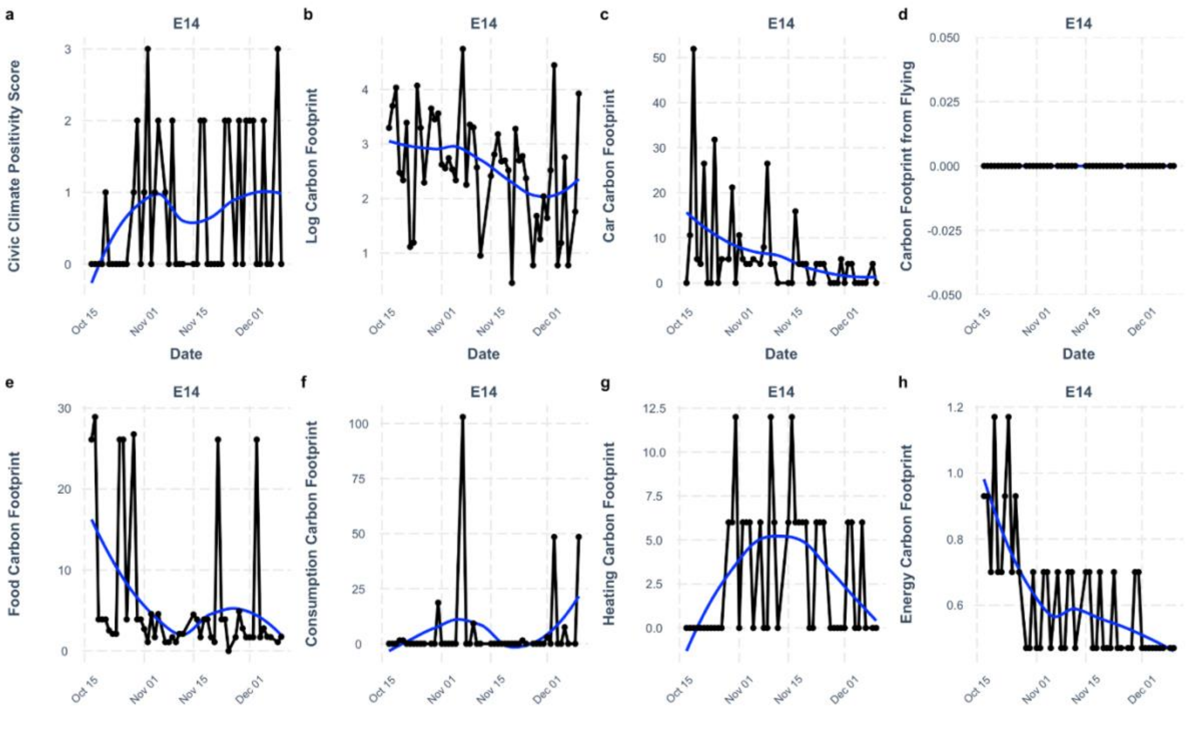

Supplementary Figure 5.2 Dynamic behavioural profile for study participant E14 (treatment group)

Figure 5.2 presents the behavioural dynamics for participant E14 from the treatment group as an example, who took part in the study from the start and until the end. We see here for instance an increase in civic and political climate action at the start, then more inaction in the middle of the study, and then again more action towards the end. The overall log carbon footprint is fluctuating, but overall decreasing, with a slight increase again at the end. We can also see a clear decrease in the carbon footprint from car journeys. Again, this study participant did not fly during the study period. The food carbon footprint is rather high in the beginning, then drops quite substantially and remain mostly low, with two exceptions. Consumption carbon footprint goes up and down again, with another increase towards the end, just before Christmas. Heating data seems to suggest an effort to minimise heating carbon footprint, with occasional spikes in November. There is also a clear pattern of decreasing carbon

footprint from energy. At the start of the study, the participant did not write much in terms of open-text comments, but this changes after the first week. They write that they are talking increasingly to others about climate change and that they are getting increasingly informed and that as they learn more about climate change, they are increasingly in shock. They report avoiding heating (putting on jumper etc. instead) and to heat for fewer hours. They report how they try to influence others, e.g. persuaded their daughter to buy a second-hand coat. With respect to electricity, they write that they changed the washing machine setting to 30 degrees and that they are trying to reduce car usage. They also joined a climate change group on Facebook. Moreover, they checked her bank (Santander) with respect to climate change and were satisfied: “Today I looked into what actions my bank is doing to help climate change. I must admit they are looking pretty good. Santander!” This participant clearly underwent some changes over the course of the study.

With these two examples we want to highlight the potential of the data for extracting stories, pathways and trajectories of how people change or not and if they do not change, what their thoughts and rationales are. Linking this further with data on socio-demographics, attitudes, values and the rich reflections provided by participant in the exit survey, gives us unique insights into people’s everyday life and mindsets in relation to climate change and specifically climate change mitigation.

Supplementary Note 6: Overview over pre-registered hypotheses

**Hypothesis**	**Result Summary**	**Cross-Reference**
H1: Exposure to moral arguments for climate action decreases individual's carbon footprint.	Confirmed, but only in interaction with time	2.1
H2: Exposure to moral arguments for climate action increases individual's civic climate positivity score.	Confirmed	2.8
H3: Exposure to moral arguments for climate action increases overall climate positive behaviour (reduction in carbon footprint + increase in civic and political climate positive behaviour).	Confirmed, but only in interaction with time	2.9
H4: Behavioural changes in the experiment group stabilise over time, i.e. clearer direction in behavioural change and less fluctuation.	Rejected	2 and 5
H5: Study participation within the control group leads to an increase in climate positive behaviour, but the increase is lower and less stable over time in comparison to the experimental group.	Confirmed only for civic climate positive behaviour	2.8, 2
H6: Individuals, who identify as rather on the political left, will show an increase in climate positive behaviour across both control and experimental group, but the increase will be bigger in the experimental group.	Confirmed for car usage carbon footprint and food carbon footprint<br>Rejected for other carbon footprint outcomes.<br>Rejected (opposite effect) for civic climate positive behaviour	3.1
H7: Individuals, who hold altruistic values will show an increase in climate positive behaviour across both control and experimental group, but the increase will be bigger in the experimental group.	Rejected	3.2
H8: Individuals, who hold biospheric values will show an increase in climate positive behaviour across both control and experimental group, but the increase will be bigger in the experimental group.	Rejected	3.2

H9: Individuals, who perceive social norms as being overall prescriptive of climate-action and prohibitive of climate damaging behaviours, will show an increase in climate positive behaviour across both control and experimental group, but the increase will be bigger in the experimental group.	Rejected	3.3
H10: Individuals, who perceive social norms as changing towards overall being prescriptive of climate-action and prohibitive of climate damaging behaviours, will show an increase in climate positive behaviour across both control and experimental group, but the increase will be bigger in the experimental group.	Rejected	3.3.
H11: Participants in the experimental group will show greater concern about climate change after participating in the 8-weeks field experiment.	Rejected	3.4
H12: Participants from both groups, who spoke about climate change with people around them during the study, are more likely to perceive social norms being/changing to overall prescriptive of climate-action and prohibitive of climate damaging behaviours after their 8-weeks field-experiment study participation.	Not tested, separate analyses to be conducted	NA
H13: Participants with higher sense of agency are more likely to show climate positive behaviour (lower carbon footprint and higher civic climate positivity score).	Confirmed for civic climate positivity score	3.5
H14: interaction effect between feeling anxious or distressed about climate change/ecological issues and efficacy perceptions effects predict climate positive behaviour	Rejected	3.6
H15: People, who own the property they are inhabiting, are more likely to have a green heating system or consider installing one as a result of participating in the study.	Could not be tested, as sparse data on green heating system	NA
H16: People with higher disposable household income, are more likely to own an electric vehicle.	Could not be tested, as sparse data on EVs	NA
H17: People with higher disposable household income, have a higher carbon footprint.	Confirmed	3.5, 3.6
H18: Female participants are more likely to adhere or shift to a predominantly plant-based diet over the course of the study.	Rejected	3.5, 3.6

See pre-registration, https://doi.org/10.17605/OSF.IO/XAEKZ

Supplementary References

Akenji, L., Bengtsson, M., Toivio, V., Lettenmeier, M., Fawcett, T., Parag, Y., Saheb, Y., Coote, A., Spangenberg, J., Capstick, S., Gore, T., Coscieme, L., Wackernagel, M., Kenner, D. (2021): 1.5-Degree Lifestyles: Towards A Fair Consumption Space for All. Hoot or Cool Institute. URL: https://hotorcool.org/wp-content/uploads/2021/10/Hot_or_Cool_1_5_lifestyles_FULL_REPORT_AND_ANNEX_B.pdf

Bouman, T., Steg, L., Kiers, H.A.L. (2018): Measuring Values in Environmental Research: A Test of an Environmental Portrait Value Questionnaire. Frontiers in Psychology, 9, 564, https://doi.org/10.3389/fpsyg.2018.00564
